# Unlocking the genetic diversity and population structure of the newly introduced two-row spring European HerItage Barley collecTion (ExHIBiT)

**DOI:** 10.3389/fpls.2024.1268847

**Published:** 2024-03-20

**Authors:** Villő Bernád, Nadia Al-Tamimi, Patrick Langan, Gary Gillespie, Timothy Dempsey, Joey Henchy, Mary Harty, Luke Ramsay, Kelly Houston, Malcolm Macaulay, Paul D. Shaw, Sebastian Raubach, Kevin P. Mcdonnel, Joanne Russell, Robbie Waugh, Mortaza Khodaeiaminjan, Sónia Negrão

**Affiliations:** ^1^ School of Biology and Environmental Science, University College Dublin, Dublin, Ireland; ^2^ School of Agriculture and Food Science, University College Dublin, Dublin, Ireland; ^3^ Cell and Molecular Sciences, The James Hutton Institute, Dundee, United Kingdom; ^4^ Department of Information and Computational Sciences, The James Hutton Institute, Dundee, United Kingdom; ^5^ School of Biosystems Engineering, University College Dublin, Dublin, Ireland; ^6^ Division of Plant Sciences, University of Dundee at The James Hutton Institute, Dundee, United Kingdom

**Keywords:** barley, genetic resources, agronomic characterization, germplasm collection, genome-wide association studies, plant phenotyping

## Abstract

In the last century, breeding programs have traditionally favoured yield-related traits, grown under high-input conditions, resulting in a loss of genetic diversity and an increased susceptibility to stresses in crops. Thus, exploiting understudied genetic resources, that potentially harbour tolerance genes, is vital for sustainable agriculture. Northern European barley germplasm has been relatively understudied despite its key role within the malting industry. The European Heritage Barley collection (ExHIBiT) was assembled to explore the genetic diversity in European barley focusing on Northern European accessions and further address environmental pressures. ExHIBiT consists of 363 spring-barley accessions, focusing on two-row type. The collection consists of landraces (~14%), old cultivars (~18%), elite cultivars (~67%) and accessions with unknown breeding history (~1%), with 70% of the collection from Northern Europe. The population structure of the ExHIBiT collection was subdivided into three main clusters primarily based on the accession’s year of release using 26,585 informative SNPs based on 50k iSelect single nucleotide polymorphism (SNP) array data. Power analysis established a representative core collection of 230 genotypically and phenotypically diverse accessions. The effectiveness of this core collection for conducting statistical and association analysis was explored by undertaking genome-wide association studies (GWAS) using 24,876 SNPs for nine phenotypic traits, four of which were associated with SNPs. Genomic regions overlapping with previously characterised flowering genes (HvZTLb) were identified, demonstrating the utility of the ExHIBiT core collection for locating genetic regions that determine important traits. Overall, the ExHIBiT core collection represents the high level of untapped diversity within Northern European barley, providing a powerful resource for researchers and breeders to address future climate scenarios.

## 1 Introduction

Food security is a pressing global issue, with agricultural production facing severe yield penalties due to abiotic stresses caused by climate change (Prosekov & Ivanova, 2018; Masson-Delmotte et al., 2021; Sasidharan et al., 2021). Barley is the fourth most produced cereal crop, and is used for human food, animal feed and malting alcoholic drinks. Therefore, it is a key crop for food security as it can be grown in many marginal communities where very few other crops survive (Newton et al., 2011).

Barley was domesticated around 10,000 years ago in the Fertile Crescent (Badr et al., 2000) and has since undergone continuous selection and breeding. Traditional breeding methods and recent advances in genetic engineering have significantly increased barley yields (Harwood, 2019). However, due to past population bottlenecks, genetic drift, and inbreeding, modern barley cultivars have a narrow genetic basis compared to landraces and wild ancestors (Schmidt et al., 2023). This has resulted in modern cultivars becoming more susceptible to stress and less adaptable to changing environments (Tanksley and Mccouch, 1997; Caldwell et al., 2006). Landraces and cultivars bred before the Green Revolution exhibit improved stress resilience and adaptation to their environments (Slama et al., 2018; Marone et al., 2021), but their exceptional genetic potential remains largely uncharacterized (Newton et al., 2010, 2011; Monteagudo et al., 2019).

Natural genetic diversity is a key pillar of plant breeding, with most breeding techniques heavily relying on it as the canvas for breeders to improve and develop new cultivars. To screen for genetic diversity, it is important to have a diverse collection with a large number of accessions. Collections with higher levels of diversity are more likely to harbour resilience, that has of yet remained untapped. In addition, in association methods such as genome-wide association studies (GWAS), variation is essential for the establishment of a relationship between markers and traits (Korte and Farlow, 2013). However, phenotyping of a large collection can be costly and time consuming, and some accessions can be highly similar, resulting in additional work without the associated benefits (Frankel and Brown, 1984; Berger et al., 2012; Araus et al., 2018). The core collection strategy minimises repetitiveness within a collection while preserving genetic diversity and reducing phenotyping costs (Brown, 1989a, b). To establish a core collection, highly similar accessions within the collection need to be identified and removed, which can be achieved using the passport data of the accessions, population structure and phenotypic characterization of the collection. The barley research community has established several core collections to study diversity and evolution as well as to screen for biotic and abiotic stress responses. For instance , the International Barley Core Collection (BCC) was established in 1989 (van Hintum, 1994; van Treuren et al., 2006) to reflect the diversity of barley worldwide, and since its inception, it has been used to better understand barley‘s diversity and evolution (Liu et al., 1999, 2000, 2001, 2002). The Spanish Core Collection was assembled to show the diversity of barley found within the Spanish Germplasm Bank (Igartua et al., 1998), and the Czech winter barley collection (Dreiseitl, 2021; Dreiseitl and Nesvadba, 2021) has been recently established to screen for disease resistance.

The majority of barley collections have been primarily focused on accessions from Southern Europe, disregarding the specific needs and challenges of barley production in Northern Europe (Pasam et al., 2014; Selçuk et al., 2015). In Northern Europe, which accounts for 25% of the EU’s malting production capacity (Euromalt, 2021), the impact of climate change on cultivation is becoming increasingly challenging. Malting is the premium use product for barley (Hertrich, 2013), with two-row spring barley being the main target of selective breeding for malting quality and yield (Tondelli et al., 2013). The growing craft brewing (Guido, 2019) and distilling markets (Umego and Barry-Ryan, 2022) have created an increased demand for barley with favourable malting quality and unique taste. The Northern European region is expected to experience deteriorating agricultural conditions due to climate change, characterized by more frequent extreme weather events, excessive precipitation, and even drought (Uleberg et al., 2014; Nolan and Flanagan, 2022), leading to a reduction in barley production (Xie et al., 2018). Assembling, characterising and utilising genetic resources capable of overcoming these threats will help ensure the resilience and productivity of barley, even in changing climatic conditions. Moreover, fostering barley production using low carbon emission methods will enhance the sustainability of the critically important malting industry (European Commission, 2020; Sleight, 2022).

This study aims to: (i) assemble a natural and diverse two-row spring barley collection (ExHIBiT) focusing on Northern European accessions and investigate its genetic and phenotypic diversity (ii) establish a core-collection of two-row spring barley for multiple purposes, and (iii) analyse the role of geographic origin and breeding history in the formation of the ExHIBiT genetic structure using the 50k iSelect SNP array (Bayer et al., 2017). The ExHIBiT collection is predominantly composed of Northern and Central European accessions due to their historical contribution for the malting industry in these regions. Characterisation of the ExHIBiT collection will promote the use of heritage barley as an untapped reservoir of genetic variation for breeders and support the identification of quantitative trait loci (QTL), facilitating the advance genetic and breeding research and tackling barley sustainability in Northern and Central Europe.

## 2 Materials and methods

### 2.1 Plant material

The ExHIBiT collection comprised of 363 two-row spring barley accessions, from several gene banks and collections, namely from the Department of Agriculture, Food and the Marine (DAFM), Ireland, Nordic Genetic Resource Centre (NordGen), Norway, the James Hutton Institute (JHI), UK. The germplasm material from JHI includes accessions from i) Bustos-Korts et al., 2019, namely from the Wheat and barley legacy for breeding improvement (WHEALBI) project (https://www.whealbi.eu/); ii), IMPROMALT project (Looseley et al., 2020); iii) 9k project and Heritage collection (Schmidt et al., 2019), as well as the Germplasm Resource Unit of the John Innes Centre (GRU-JIC) and JHI stocks. Accessions were selected based on their passport information, to reflect their genetic diversity and breeding history. Particular focus was placed on Northern and Central European accessions, taking into consideration previous population structure results of a European two-row spring barley collection (Saade et al., 2016). In this work, the term heritage is used to encompass the intricate crop breeding history of a region, which is in turn influenced by historic and ever-changing factors such as source material, climate, land type, evolving agricultural equipment, manufacturing processes and market demands. We further characterise these endemic resources into three groups. (i) Landraces: highly diverse material continually selected by producers over time and therefore well adapted to local environments; (ii) Old Cultivars: cultivars actively selected by formal breeding programs prior to the Green Revolution (Pre-1960s) and; (iii) Elite Cultivars: cultivars developed in the modern era of plant breeding (post-1960) and after the introduction of the Distinctiveness, Uniformity and Stability (DUS) testing in the United Kingdom. The completed list of accessions, including year of release and geographical origin based on information from JHI, the European search catalogue for plant genetic resources (EURISCO) and previously published works (Maxted et al., 2014; Weise et al., 2017; Faccini et al., 2021) are given in **Supplementary Table S1**.

### 2.2 Genotyping of the ExHIBiT collection

DNA was extracted from an individual plant utilising two-week-old leaf tissue of the accessions within the ExHIBiT collection using the DNeasy Plant Mini Kit (Qiagen, Germany) following the manufacturer‘s protocol. Plants for DNA extraction were grown in controlled conditions prior to any field experiment. Accessions were genotyped at the JHI using the Illumina Infinium *iSelect* HD 50k chip, which was designed to capture the most representative set of barley germplasm (Bayer et al., 2017). Physical positions of markers were based on the pseudo-molecule assembly of the most recently updated barley reference assembly- “Morex” V3 (Mascher et al., 2021). Markers with a call rate value lower than 90% and minor allele frequency (MAF) lower than 5% were removed using TASSEL (Bradbury et al., 2007). To identify any duplicate lines within the collection, standard R v3.6.0 0 (R Core Team, 2022) was used to calculate the similarity between the accessions by examining the percentage of markers sharing the same nucleotide. The SNP marker data for this study have been deposited in the European Variation Archive (EVA) at EMBL-EBI under accession number PRJEB67728 (https://www.ebi.ac.uk/eva/?eva-study=PRJEB67728).

### 2.3 Population structure and pedigree analysis

To determine ExHIBiT population structure, genotypic data was analysed using STRUCTURE V.2.3.4 software, which uses a Bayesian clustering approach to assign individuals to K subgroups (Pritchard et al., 2000). Five independent runs (K =1 to 10) were performed with 50,000 burn-in periods, and 10,000 Markov Chain Monte Carlo iterations for each value of K. The best number of K was chosen using the ΔK method (Evanno et al., 2005) by running the Structure Harvester software (Earl and VonHoldt., 2012). Accessions were classified as belonging to a group if more than 50% of the markers belong to that group, otherwise they were classified as admixture.

To construct the Neighbour Joining Tree (Saitou and Nei, 1987) from the 363 barley accessions, using simple matching of markers, the R package APE: Analysis of Phylogenetics and Evolution (Paradis et al., 2004) was employed. The phylogenetic tree was visualised using R package phytools (Revell, 2012). Phylogenetic distances between accessions were calculated using R package ‘adephylo’ (Jombart et al., 2010). Principal Component Analysis (PCA) was conducted on the same set of data using R package pcaMethods (Stacklies et al., 2007) and visualised using R package ggplot2 (Wickham, 2016).

Pedigree data of the collection was gathered from various sources including historical records held at JHI, breeder supplied pedigree definitions, manuscripts and other written communications, pedigree definitions obtained from the Agriculture and Horticulture Development Board (AHDB) pocketbooks and finally the AHDB Recommended Lists app (https://ahdb.org.uk/knowledge-library/recommended-lists-for-cereals-and-oilseeds-rl-app). The collated data was checked for inconsistencies between data sources and finally formatted in Helium format files that can be visualised using Helium (https://helium.hutton.ac.uk), where pedigree structure can be explored and additional data types uploaded and overlaid on the pedigree for visualisation and analysis.

### 2.4 Phenotyping the ExHIBiT collection

The ExHIBiT collection was studied during 2020 under field conditions at University College Dublin (UCD) Lyons Estate Research Farm, Ireland (53.18322, -6.31398) along with three checks, namely Golden Promise (top old cultivar in Europe), RGT Planet (top cultivated elite cultivar in Europe in 2020’s) and Propino (top cultivated elite cultivar in Europe in 2010’s). Checks were used to detect and correct for spatial variation across the trial blocks, ensuring that the partial replication provided an estimate of the trial error. The field was divided into 15 rows and 30 columns, which formed a grid of 50 blocks with nine (3 by 3) plots in each block. Each plot contained a primary check (RGT planet) at its centre and a secondary check (RGT planet, Golden Promise or Propino) was randomly placed around the field. In total, 52 RGT planet, 17 Propino and 16 Golden Promise plost were used. Each accession plot measured 4m by 0.45m, containing four rows of plants with 15 cm spacing between them. Accessions were grown according to local management practices in terms of sowing rate, weed and disease control, and fertiliser inputs. The sowing rate of 140.8 kg Ha-1 was maintained consistently across all accessions following the recommendations of Teagasc, the Agricultural and Food Development Authority of Ireland. A full outline of the trial dates, fertilisation and weed control practices are provided in **Supplementary Table S2**. Description of weather including temperature, relative humidity and precipitation during the growing season is presented in **Supplementary Table S3**. During sowing, the accessions with ID number from 338 to 363 in the last row of the field were phenotypically unreliable due to sowing equipment malfunction; hence, phenotypic data for these accessions was not collected and row-type was labelled as unknown. However, despite the lack of their phenotypic data, the accessions 338-363 were included in the genotypic analysis to investigate the overall genetic variability of the ExHIBiT collection as this information was collected prior to field trials.

A total of nine phenotypic traits were recorded for each accession (**Table 1**), with the timing of main stages being recorded according to the Zadoks growth stage scale (Zadoks et al., 1974). Flowering time (FLT) was recorded according to Alqudah and Schnurbusch (2017). During the harvest, plot samples were manually cut at ground level from one of the middle rows (linear metre) and stored in a glasshouse prior to processing. To determine the Shoot Fresh Mass (SFM), grain yield (YLD), and harvest index of each accession, the samples were threshed and cleaned using machinery from Almaco, Nevada, USA. The machinery used included the thresher model SBT (serial number 99005) and the seed cleaner model ABSC (serial number 99006). The harvest index (HI) is defined as the ratio of harvested grain to total shoot dry matter. The spikes of all accessions were photographed to create a spike image library which is available from the ExHIBiT Germinate (Raubach et al., 2021) database (http://ics.hutton.ac.uk/germinate-exhibit).

**Table 1:** List of nine traits recorded during the 2020 and 2021 field trials. Includes type of trait, name of trait, method of measurement and unit of measurement.

	Trait	Abbreviation	Method of measurement	Unit
Pre-harvest traits	Tiller count	TN	Number of tillers per plant (average from three plants per plot)	–
	Flowering time	FLT	Number of days from sowing to flowering	days
	Ripening period	RIP	Number of days from sowing to ripening	days
	Height	HEI	Distance from soil surface to tip of the spike (excluding awns), averaged from four measurements	cm
Post- harvest traits	Shoot Fresh Mass	SFM	Weight of fresh shoot per linear metre	kg
	Grain Yield	YLD	Weight of grains per linear metre	g
	Harvest Index	HI	Ratio between grain yield (YLD) and shoot fresh mass (SFM)	%
Seed traits	Thousand kernel weight	TKW	Weight of 1000 kernels	g
	Protein content	PRO	Protein content of seeds after drying	%

### 2.5 Power analysis and selection of ExHIBiT core collection

To reduce the number of accessions while preserving the diversity of the collection, the ExHIBiT core collection was established using a power analysis, in which the number of accessions was determined to achieve sufficient association power using both genotypic and phenotypic data (which included all recorded traits from 2020 except Protein Content-PRO). Effective sample size and statistical power was computed using the R package “pwr” (Champely, 2020). The power and sample sizes were calculated under different ranges of factors, including MAF of 0.5, 0.2, 0.01. The core collection was set up by identifying the most diverse genotypes using the Core Hunter 3 software (De Beukelaer et al., 2018), in which subsets on the bases of multiple genetic and phenotypic measures, including both distance measures and allelic diversity indices were assembled. To further confirm the conservation of genetic diversity in the core collection, the results of the genetic principal components (PCs) of the whole ExHIBiT collection were compared to the PCs of the core collection. Wilcox test was performed by running the wilcox.test r function from R package stats v4.2.2 (R Core Team, 2022) between the phenotypic data from 2020 of the whole ExHIBiT collection versus the core collection.

### 2.6 Phenotyping the ExHIBiT core collection

A total of 230 accessions comprising the ExHIBiT core collection, along with two checks (RGT planet and Golden Promise), were trialled at UCD Lyons Estate Research Farm in 2021. The experimental layout consisted of two blocks arranged in a completely randomized design. The core collection was fully replicated, and checks were randomly replicated across the field, in summary each block included a fully replicated instance of the ExHIBiT core collection and 22 replicates of each check randomly distributed. Trial dates, fertilisation and weed control practices together with weather conditions during growth season are provided in **Supplementary Table S2, S3**, respectively. The sowing rate of 140.8 kg Ha-1 was maintained consistently across all accessions. The field trial comprised a total of 504 plots, distributed by 24 rows and 21 columns. The plot size was 7m by 0.60m, containing 5 rows of plants with a row spacing of 0.15m; thus, enabling the retrieval of agronomic data. For sowing, the Wintersteiger (A-4910 Reid, Austria machinery with the serial number 2270-4014-PDS-E and the machinery type Plotseed XL) was used. The same traits were recorded as previously described for 2020 (**Table 1**).

### 2.7 Statistical analysis of phenotypic data

An initial step of data cleaning and processing included outlier removal (for both 2020 and 2021) using Tukey’s method (Anscombe and Tukey, 1963) with outliers removed from both within and across the years according to Khodaeiaminjan et al. (2023). The data was then adjusted for the spatial variation in the field using a mixed-model analysis for each trait in each year using ASReml-R v4.0 (Butler et al., 2017) and asremlPlus (Brien, 2023) packages for the R statistical computing environment R v3.6.0 (R Core Team, 2022). In brief, the formula of the maximal mixed model for this analysis is:


y = Xβ + Zu + e;

where y is the vector of values of the trait analysed and β, u and e are the vectors for the fixed, random, and residual effects, respectively. The design matrices corresponding to ‘β’ and ‘*u’* are denoted by X and Z, respectively. The checks, blocks, position, and genotype effects were all accounted for in this model. To identify the environmental terms that were sources of variation and needed to be included in the analysis model, variograms were examined following recommendations outlined by Gilmour et al. (1997). From these analyses, the best linear unbiased estimates (BLUEs) were obtained and used as an input for the subsequent association analysis. The heritability was calculated according to Cullis et al. (2006). Full details about the spatial correction of the field data can be found in Saade et al. (2016).

Correlation analysis was performed using the Pearson correlation with R package Hmisc (Harrell, 2023), and the correlation matrix figure was generated with R package corrplot (Wei and Simko, 2021). PCA of the phenotypic data was conducted using R package pcaMethods (Stacklies et al., 2007) and visualised using R package ggplot2 (Wickham, 2016). All scripts used are available in Germinate at http://ics.hutton.ac.uk/germinate-exhibit.

### 2.8 Genome-wide association studies

GWAS was performed using the Genome Association and Prediction Integrated Tool package (GAPIT) (Wang and Zhang, 2021). Bayesian-information and Linkage-disequilibrium Iteratively Nested Keyway (BLINK), a state-of-the-art multivariate model was employed for GWAS as a suitable model for smaller populations (~200) (Huang et al., 2019). To cope with population structure, kinship matrix and PCs were included in the model. The optimal number of PCs for each trait was determined using the results from the population structure analysis, and by analysing quantile-quantile (QQ) plots, created by the qqPlot() function in the “car” package in R (Fox and Weisberg, 2019), which are commonly used to effectively determine false positives and negative associations (Riedelsheimer et al., 2012; Kristensen et al., 2018). False discovery rate (FDR<0.05) was considered as the significant association threshold between markers and traits (Storey and Tibshirani, 2003; Storey et al., 2022), together with the Bonferroni-adjusted threshold of α = 0.05. Phenotypic distribution of significant SNPs identified in GWAS was analysed using t-test.

### 2.9 Linkage Disequilibrium (LD) analysis and candidate gene selection

Pairwise Linkage disequilibrium (LD) analysis was carried out according to Khodaeiaminjan et al. (2023). In short, LD-decay and LD-blocks were analysed using the ‘Ldheatmap’ R package (Shin et al., 2006) for each chromosome. Regions of interest for candidate genes were considered: i) genome region containing a significant marker in which flanking markers displayed strong LD (r2>0.5), and neighbouring markers on either side and ii) genome region containing significant markers outside of LD block defined by flanking marker. The allelic diversity of the SNP markers previously identified by Bustos-Korts et al. (2019) was examined by quantifying the percentage makeup of two alleles at those SNP regions.

## 3 Results

**Figure 1:**
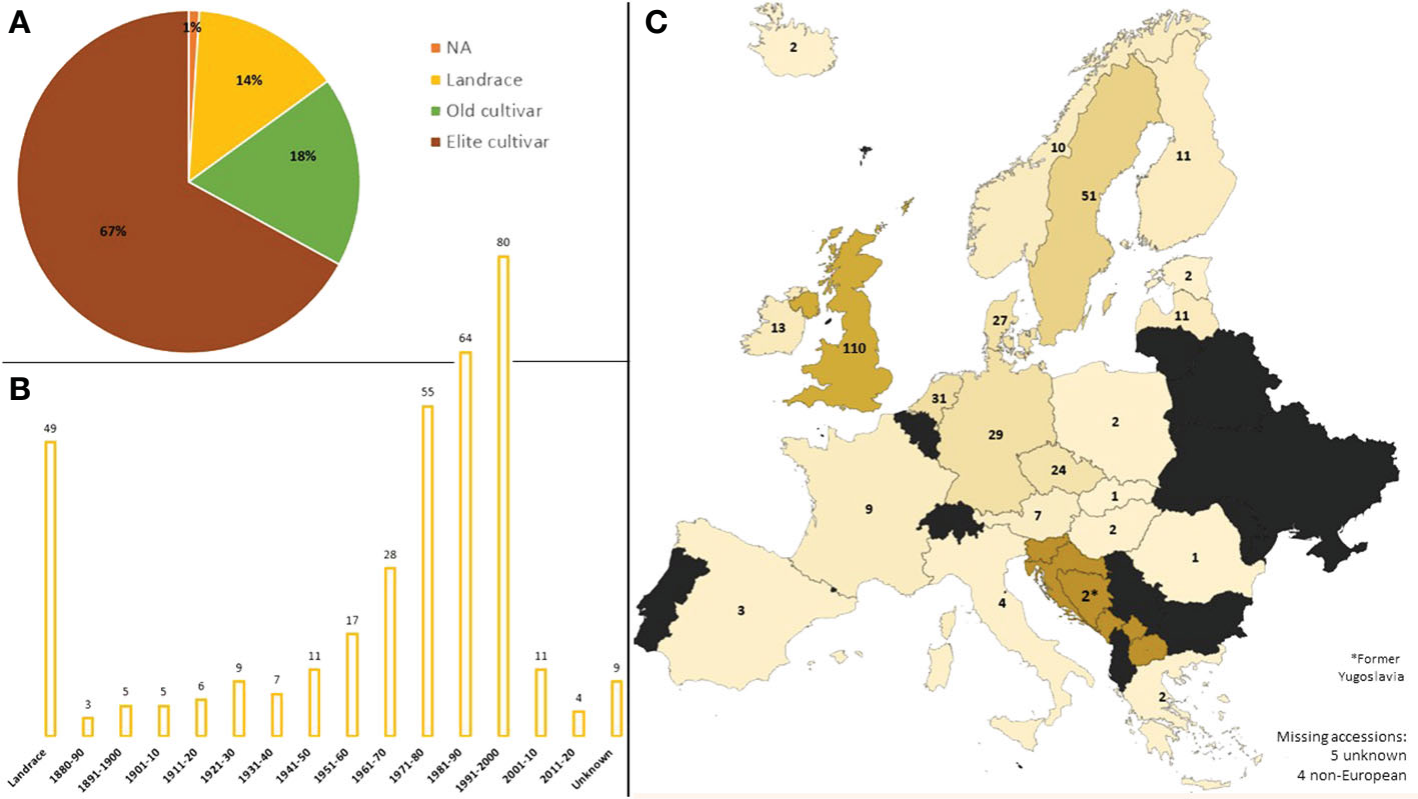
The Background of the ExHIBiT collection. **(A)** Breeding history of accessions in the ExHIBiT collection, divided into landraces, old cultivars (released before 1960) and elite cultivars (released after 1960). **(B)** The decade of release of accessions in the ExHIBiT collection, showing the number of accessions released in each decade in the collection. Landraces are separated as they do not have a specific year of release. **(C)** Country of origin of the accessions in the ExHIBiT collection. In the case of landraces, this is the country where the accession was found. In the case of the old and elite cultivars, this is the location of the institute or company where breeding took place. The origin of one accession is unknown and four lines originate from outside of Europe.

The ExHIBiT collection, comprising 363 barley accessions, from 22 European countries (including the former Yugoslavia) was assembled in this study. The collection specifically focused on Northern Europe (70%), with most accessions coming from the UK (30%). Four accessions originate from outside of Europe due to mislabelling in genebanks. The ExHIBiT collection includes elite cultivars (~67%), old cultivars (~18%), landraces (~14%), and accessions with unknown breeding history (~1%), representing the genetic diversity and breeding history of two-row spring barley in Europe, with the majority of accessions being released before the 90’s (**Figure 1**). Full passport data for genotypes (based on information from JHI, the European search catalogue for plant genetic resources (EURISCO), Weise et al. (2017) and Faccini et al. (2021) is provided in **Supplementary Table S1**. Despite only accessions labelled as ‘two-row’ types being selected for this study, 16 out of the 363 accessions in the ExHIBiT were identified as six-row upon phenotypic analysis. These accessions remained as part of the ExHIBiT collection but were not considered for inclusion in the core collection and not included in subsequent studies. The entire collection was genotyped using the 50k iSelect SNP array (Bayer et al., 2017). In total 35,968 markers were mapped to a physical position on the “Morex” V3 genome sequence (Mascher et al., 2021). Full 50k SNP array data for the collection can be found on the germinate website (http://ics.hutton.ac.uk/germinate-exhibit). After MAF and missing data filtering 26,585 robust markers remained for genotypic analysis. This data set is available at EMBL-EBI under accession number PRJEB67728 (https://www.ebi.ac.uk/eva/?eva-study=PRJEB67728) (**Supplementary Figure S1**).

### 3.1 Phylogenetic relationship & population structure of the ExHIBiT collection

**Figure 2:**
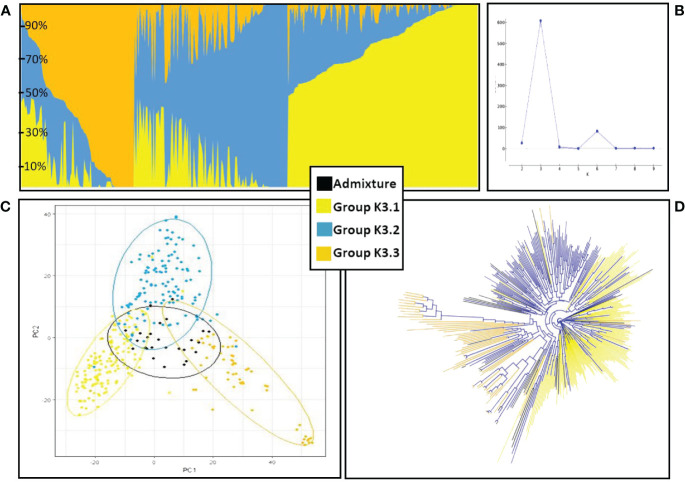
Population structure and phylogenetic analysis of ExHIBiT collection. **(A)** Results of structure analysis for K=3, each vertical line represents an accession, y-axis shows percentage content of each accession to the three different groups. **(B)** Graph of Delta K values for K 2 to K 10. Maximum DeltaK was reached at K = 3 and another peak at K=6. **(C)** Principal Component Analysis based on 50K genotypic data coloured according to the three groups identified in population structure analysis. **(D)** phylogenetic tree coloured according to the three groups identified in population structure analysis.

**Figure 3:**
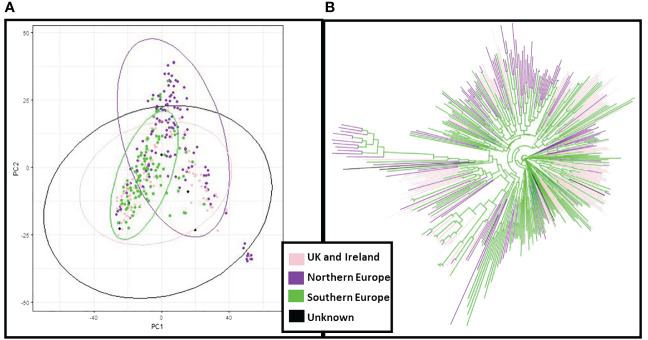
Principal Component Analysis (PCA) and neighbour joining phylogenetic analysis based on the region of origin of the ExHIBiT collection. **(A)** PCA coloured by the region of origin **(B)** phylogenetic tree coloured by region of origin. In pink shown accessions from the UK and Ireland, in purple accessions from Northern Europe, in green accessions from Southern Europe and in black accessions whose region of origin is unknown.

The genetic structure of the ExHIBiT collection showed three main groups (**Figure 2A**), which can be further divided into six smaller sub-groups, as shown by the ΔK peaks at K3 and K6 (**Figure 2B**). The fixation index (Fst) showed significant divergence within the groups, with values of 0.32, 0.48 and 0.35 for three groups of K3.1, K3.2, and K3.3 respectively. K3.1, K3.2, K3.3 contained 151, 122 and 64 accessions respectively, with the remaining 26 accessions being classified as admixture. These groups can also be distinguished in both PC & phylogenetic trees (**Figures 2C, D**). The division of these three groups was investigated using geographical data in three main regions of origin (UK and Ireland, Northern and Southern Europe) (**Figures 3A, B**). However, the results showed that the population structure was not influenced by geographical origin. To further explore the ExHIBiT population structure, breeding history was investigated, and the results showed that in K3.1, 56% of the accessions were released post-1990 and 43% pre-1990. While in K3.2, 86% of the accessions had been released pre-1990 and in K3.3, 63% of accessions were landraces according to known breeding history. In these six groups, the K3.3 group split into K6.1 and K6.2 where K6.1 contains the two-row landraces and K6.2 all the six-row landraces (**Supplementary Figure S4**). In the population structure analysis, the accessions categorised as “admixture” were predominantly elite cultivars, comprising approximately 69% of the group. The remaining 31% of this category was divided into 12% landraces and 19% old cultivars. This distribution closely mirrors the overall composition of the collection.

**Figure 4:**
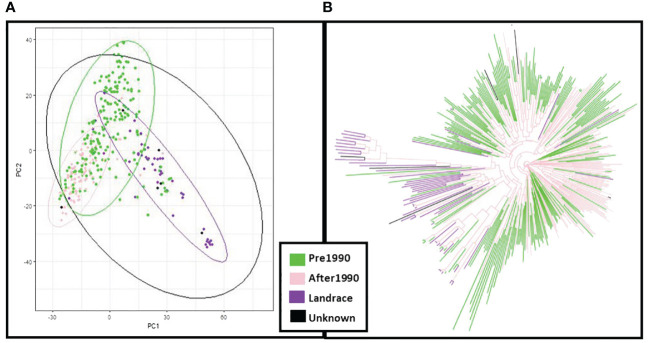
Principal Component Analysis (PCA) and neighbour joining phylogenetic analysis based on the year of release of the ExHIBiT collection **(A)** PCA coloured by year of release. **(B)** phylogenetic tree coloured by year of release. In green shown accessions released before 1990, in pink accessions released after 1990, in purple accessions landraces and in black accessions whose year of release is unknown.

In principal component analysis of genotypic data, PC1 and PC2 explained 8% and 6% of variation respectively. Clustering by PCA and phylogenetic analysis (**Supplementary Figure S2**) results are consistent with a population structure of three groups representing barley breeding history (**Figures 4A, B**), with landraces, pre-1990 and post-1990 accessions being distinct. PCA confirms the distinction between the different row types with two- and six-row accessions clearly clustering away from each other (**Supplementary Figure S3**), and the six-row accessions perfectly overlapping with cluster K6.2 when analysing the PCA results according to population structure with six sub-groups (**Supplementary Figure S4**). The splitting of K3.2 into subgroups K6.3 and K6.4, and K3.3 into K6.5 and K6.6, is unclear, with no distinct overlap to either geographical origin or year of release.

**Figure 5:**
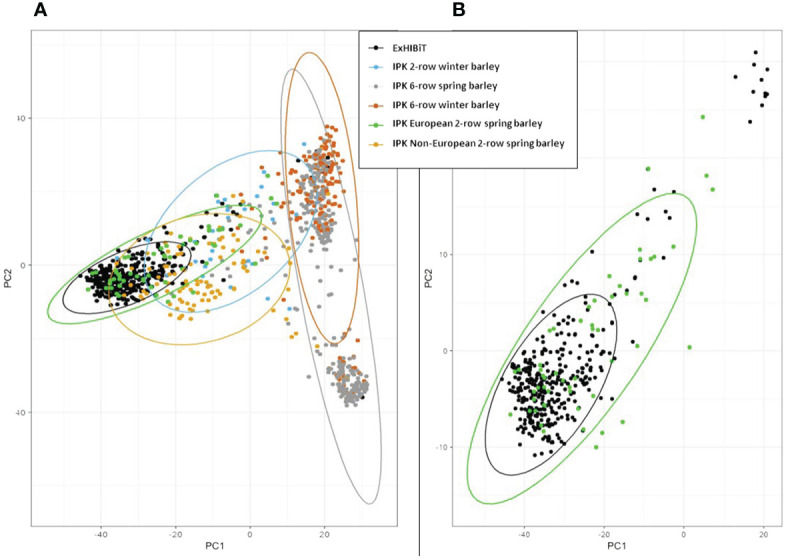
Principal Component Analysis (PCA) of ExHIBiT collection against IPK Barley Core 1000 collection. **(A)** PCA of the ExHIBiT collection (in black) clustered against the IPK Barley Core 1000 collection with European 2-row spring barley accessions from the IPK collection shown in green, non-European two-row spring barley accessions in yellow, six-row spring barley accessions in orange, two-row winter barley accessions in blue and six-row winter barley in grey. **(B)** PCA of ExHIBiT collection (in black) clustered with only the European two-row spring barley accession from the IPK Barley Core 1000 shown in green.

To assess how effectively the ExHIBiT collection captures the genetic diversity of European two-row spring barley, the genotypic diversity of the collection was compared to wider IPK Barley Core 1000 collection (Milner et al., 2019). The IPK collection reflects worldwide barley diversity and contains a large number of European accessions. Principle component analysis results combining the two collections revealed that ExHIBiT distinctly clusters with the European two-row spring barley accessions within the IPK collection (**Figure 5A**) with PC1 explaining 13% and PC2 8% of the variation in the data (**Figure 5B**).

The pedigree of lines within the ExHIBiT collection overlap with the pedigree data assembled from historical records held at JHI and supplemented with breeder declared pedigrees from AHDB and genotypes maintained at JHI for 1,847 European barley varieties (https://helium.hutton.ac.uk). The pedigree data for ExHIBiT can be visualised using the Helium pedigree visualisation platform (Shaw et al., 2014; https://helium.hutton.ac.uk/#/pedigree/exhibit) where along with pedigree definitions, additional characterization data related to the ExHIBiT collection is openly available.

### 3.2 Phenotypic variation of the ExHIBiT collection

**Figure 6:**
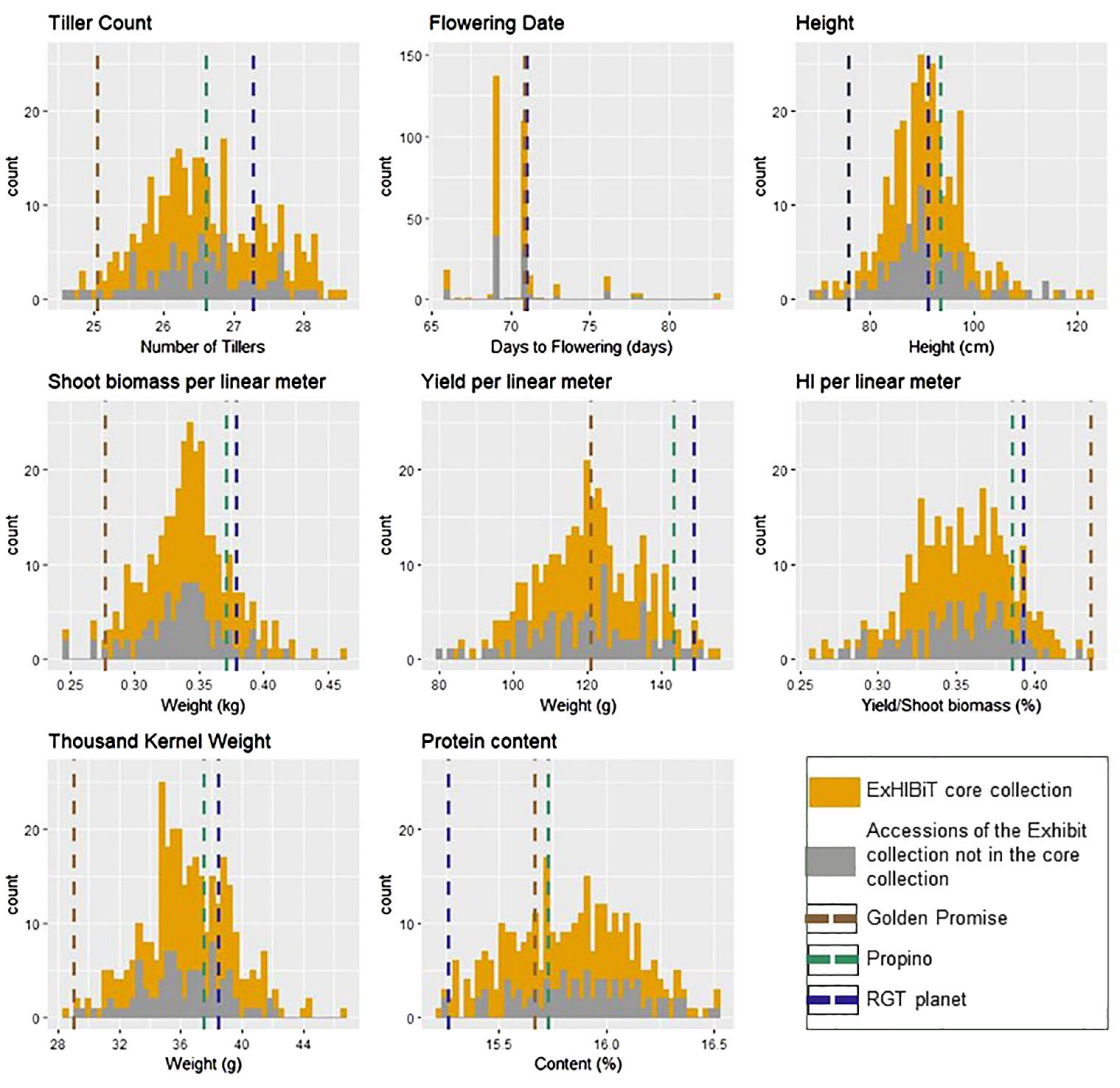
Histogram presenting the diversity difference between two sets of accessions in the 2020 field trials. The ExHIBiT core collection, consisting of 230 accessions, is represented in yellow, while the ExHIBiT collection accessions not included in the core collection, totalling 123 accessions, are shown in grey. The dashed lines represent the average values of the checks: RGT planet (in black), Propino (in green), and Golden Promise (in brown).

Nine agronomic traits were measured (**Table 1**) for all accessions in the ExHIBiT collection, in 2020. The collection showed considerable diversity in the field, with plant height (HEI) varying from 69.3cm (Kria) to 122.9cm (Irish Goldthorpe) with an average HEI of 90.6 cm. The YLD per linear metre varied from 79.8g (Craigs Triumph) to 155g (Primus) with an average of 120g (**Supplementary Table S5**). In 2020 flowering time (FLT) and ripening period (RIP) data collection was limited by weather conditions and lockdown restrictions associated with COVID19, resulting in unavoidable missing information. RIP particularly suffered from this resulting in data with nearly no variation, therefore this trait was excluded from any further analysis (**Supplementary Table S4**). The results indicate that FLT spanned from 66 to 83 days from sowing to flowering with an average of 70 days. After a visual analysis of the histograms (**Figure 6**), all traits, except FLT and RIP, appear to be normally distributed. To further illustrate the phenotypic diversity of the collection, a photo library showing the variability in shape and size of the spikes, has been included with the genotypic data on the Germinate database (http://ics.hutton.ac.uk/germinate-exhibit), and is exemplified in **Supplementary Figure S5**.

### 3.3 Construction and phenotypic characterization of the ExHIBiT core collection

To assemble a representative ExHIBiT core population, a power analysis was undertaken using phenotypic and genotypic data from the 363 accessions. Power analysis revealed that genetic effect would be detectable with 230 accessions at a power of 0.8. Regarding phenotypic data from 2020, it was observed that there was no statistical difference between the entire ExHIBiT and core collection (363 vs. 230 accessions) with *p*-values ranging from 0.96 to 0.20. The range of phenotypic values between the core collection and the entire ExHIBiT collection is highly similar (**Figure 6**). PCA results in a near perfect overlap between the whole and core collection in terms of genetic diversity, with the exception of the six-row barley accessions which were not included in core collection (**Supplementary Figure S6**). The core collection contains accessions from 20 European countries, with around 70% coming from Northern Europe. It includes elite cultivars (~74%), old cultivars (~15%), and landraces (~10%), and accessions with unknown breeding history (1%) (**Supplementary Figure S7**). The makeup of the core collection highly resembles that of the ExHIBiT collection.

The core collection was sown in April 2021 at UCD Lyons Estate Research Farm and phenotyped during the growing season until harvesting in August. The same ten agronomic traits were recorded as in 2020, and similar phenotypic diversity was observed in both years with the full dataset presented in **Supplementary Table S6** and correlation in **Supplementary Table S8**. HEI varied from 80.3cm (Wren) to 150.7cm (Chevalier Tystofte) with an average of 105.5cm. YLD per linear meter varied from 75g (Irish Goldthorpe) to 117g (Canasta) (**Table 2**).

**Table 2:** Agronomic data from ExHIBiT core collection field traits in 2021. Shows minimum, maximum, average and standard deviation for all nine collected traits^1^.

	Trait	Unit	Minimum	Maximum	Average	Standard deviation
Pre-harvest traits	TN		6.57	9.71	7.35	0.411
	FLT	days	53.86	79.97	65.75	3.91
	RIP	days	118.94	125.83	122.98	1.33
	HEI	cm	80.3	150.65	105.51	13.05
Post-harvest traits	SFM	kg	0.2308	0.3415	0.27	0.01895
	YLD	g	75.04	116.7	97.86	8.38
	HI	%	0.259	0.458	0.356	0.0276
Seed traits	TKW	g	41.26	58.89	50.25	3.048
	PRO	%	11.8	17.7	13.95	0.909

1 See **Table 1** for list of abbreviations.

**Figure 7:**
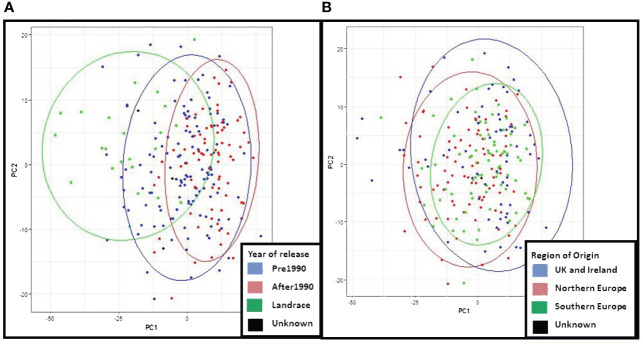
Principal Component Analysis (PCA) of 2021 phenotypic data for the ExHIBiT core collection. **(A)** PCA coloured by year of release of accessions. In green are shown landraces, in blue pre-1990 accessions and in red accession released after 1990. **(B)** PCA coloured by region of origin, in red are shown accessions from Northern Europe, in green Southern European accessions and in blue accessions from UK and Ireland.

PCA results for phenotypic data from 2021 were examined to compare to PCA results from the genotypic data and verify similar patterns in the clustering (**Figure 7**). The results show that in the PCA of 2021 phenotypic data, PC1 explained 7% of variation and PC2 3% of variation. PCA obtained from the phenotypic and genotypic data in the core collection is consistent with PCA patterns of the whole ExHIBiT collection. These results demonstrate that the region of geographical origin has no visible effect on clustering of the core collection while the year of release shows three distinct groups; landraces and accessions released before and after 1990.

Relationships between the traits and year of release were explored using correlation analysis. Correlations between years varied from 0.44 for HEI to 0.045 for SFM. Positive correlation between HEI and PRO (0.45 in 2021 and 0.16 in 2020), FLT (0.33 in 2021 and 0.36 in 2020) and SFM (0.28 in 2021 and 0.3 in 2020) were observed. Positive correlation between SFM and FLT (0.31 in 2021 and 0.14 in 2022) and YLD (0.6 in 2021 and 0.48 in 2020) were observed. Year of release negatively correlated with HEI and FLT, and positively correlated with HI and YLD (**Supplementary Figure S8**).

### 3.4 Genome-wide association studies

To confirm the value of the ExHIBiT core collection for genetic analyses, GWAS was performed on all phenotypic traits collected during the 2021 field trial (**Table 3**). The 50K SNP data was re-filtered on the core collection, resulting in 24,876 high quality markers for the 230 accessions.

**Table 3:** Genome-Wide Association Studies results for nine traits in 230 ExHIBiT core accessions using BLINK.

Trait	MAF	PCs	Associated SNP	Chromosome	Position	P.value	LD block
FLT	0.38938	3	JHI-Hv50k-2016-383902	6H	37,699,708	9.30E-08	LD Block H6 - 87
FLT	0.0996	3	JHI-Hv50k-2016-460460	7H	43,608,409	2.71E-07	LD Block H7 - 133
HEI	0.1659	3	JHI-Hv50k-2016-168497	3H	174,958,294	1.51E-10	LD Block H3 - 155
HEI	0.3362	3	JHI-Hv50k-2016-205137	3H	563,141,095	1.15E-09	LD Block H3 - 296
HEI	0.0568	3	JHI-Hv50k-2016-453491	7H	25,872,760	2.16E-07	LD Block H7 - 85
HEI	0.1288	3	JHI-Hv50k-2016-227194	4H	3,218,930	2.99E-0.6	LD Block H4 - 17
PRO	0.0526	4	JHI-Hv50k-2016-102790	2H	546,982,003	1.03E-07	LD Block H2 - 224
PRO	0.0614	4	JHI-Hv50k-2016-283903	5H	16,822,952	2.94E-0.8	LD Block H5 - 77
PRO	0.0746	4	JHI-Hv50k-2016-504314	7H	598,982,074	2.67E-10	LD Block H7 - 287
TKW	0.1288	4	JHI-Hv50k-2016-57915	1H	516,436,801	8.88E-08	JHI.Hv50k.2016.57915
TKW	0.1900	4	JHI-Hv50k-2016-117361	2H	610,739,002	1.01E-06	LD Block H2 - 287
TKW	0.0917	4	JHI-Hv50k-2016-200892	3H	545,834,674	2.91E-12	LD Block H3 - 270
TKW	0.1572	4	JHI-Hv50k-2016-358877	5H	573,661,301	1.52E-06	LD Block H5 - 492

The table includes the list of significant associations detected, the identified markers for each trait, the optimum number of PCs used for GWAS, p-values and, MAF and LD blocks are given. The positions are based on “Morex” V3 (Mascher et al., 2021).

GAPIT was used to test BLINK models. To account for the population structure, PC3 was initially tested due to the identification of three groups in the population structure analysis. After QQ plot analysis, PC4 was used as a fixed effect in BLINK whenever the results indicated that PC3 was not suitable. The QQ plots for all traits indicated that either PC3 or PC4 was appropriate, eliminating the need to run BLINK with higher PC numbers. Significant SNPs were identified for several traits: FLT, HEI, Thousand Kernel Weight (TKW), and PRO. GWAS results with Manhattan plots and phenotypic distribution of significant SNPS for HEI, TKW and PRO can be found in **Supplementary Figure S9** while the complete list of significant SNPs is presented in **Table 3**. For validation purposes, FLT was selected as an example because it is a very well described trait in barley (Alqudah et al., 2014; Bustos-Korts et al., 2019; Fernández-Calleja et al., 2021) and presented the highest heritability (0.912).

### 3.5 Genetic regions underlying FLT and diversity analysis of flowering genes

**Figure 8:**
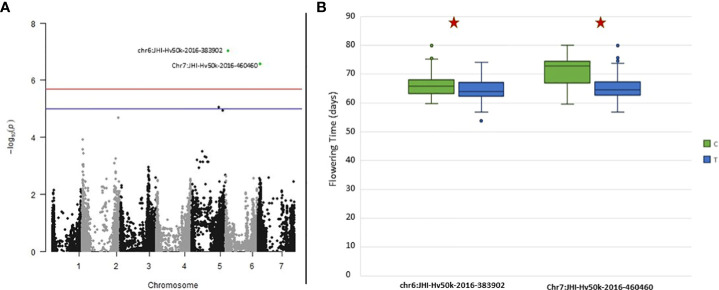
Genome-Wide Association Studies (GWAS) results for Flowering Time for the ExHIBiT core collection **(A)** Manhattan plot of GWAS analysis using BLINK with three principal components (PCs) in 2021 flowering time. In green are highlighted significant markers by Bonferroni correction (logarithm of odds (LOD) score of 5.69). In blue is the suggestive line (LOD score of 5) and in red the genome-wide significance line (LOD score of 5.5). **(B)** Boxplot showing phenotypic distribution of flowering time of two significant single nucleotide polymorphisms (SNPs) identified by GWAS with the BLINK model as observed on **(A)** results highlighted in green. Red star shows significance according to t-test (p-value< 0.05).

The GWAS results identified two markers to be significantly associated with FLT on chromosomes 6H (*JHI-Hv50k-2016-383902* located at chr6: 37,699,708) and 7H (*JHI-Hv50k-2016-460460* located at chr7: 43,608,409) (**Figure 8A**). Significant *p*-values for associations between markers and phenotypic traits were determined using the FDR (Storey and Tibshirani, 2003; Storey et al., 2022) and the Bonferroni-adjusted threshold of α = 0.05, which corresponded to a logarithm of odds (LOD) score of 5.69. Pairwise marker LD matrices and LD-decay were estimated for each of the chromosomes separately based on the SNP data showing lowest *p*-values. The LD regions expanded the genomic regions of interest to chr6:36,884,125 - 115,649,880 bp and chr7:40,532,291 - 44,676,872 bp. The significant SNPs identified on 6H overlapped with two previously identified flowering genes: *HvZTLb* (Bustos-Korts et al., 2019) and *Eam7* (Stracke and Borrner, 1998). Additionally, other flowering genes *HvZTLa*, *Vrn-H3 (*chr7:39,680,381), *HvCO8 (*chr7:50,187,671) and *HvLHY* were found to be in close proximity to the significant SNP on 7H, although they were not located within the same LD block (Alqudah et al., 2014; Bustos-Korts et al., 2019). Analysis of the phenotypic distribution of the two significant SNP markers shows statistical differences between the alleles among the population (**Figure 8B**).

To verify the genetic diversity of the ExHIBiT core collection at previously identified flowering genes, 11 flowering genes and 24 related SNP markers were examined following the findings of Bustos-Korts et al. (2019). Out of these 24 alleles, two were filtered out due to a high number of missing data. The results show that eight SNP markers within four genes have been fixed in the ExHIBiT core collection, including the *Ppd-H1* gene. The remaining 14 SNP markers and seven flowering genes showed allelic diversity in the collection. Ratio of alleles at these SNPs ranged from a near equal allelic make up (49% vs. 51% at SNP JHI-Hv50k-2016-494493 associated with gene *HvZTLa*) to one allele being predominant at SNP JHI-Hv50k-2016-411622 associated with gene *HvCO2* (13% vs. 87%).

## 4 Discussion

Here the ExHIBiT collection is introduced for the first time. The collection comprises 363 European spring barley assessions and was used to assemble the core ExHIBiT collection comprising 230 two-row accessions. This collection was created to reflect European barley diversity, and to be of use in future screening and association mapping studies to address the molecular and physiological mechanism underlying biotic and abiotic stresses.

### 4.1 Diversity & population structure of the ExHIBiT collection

Barley has a large genetic diversity, with landraces and older cultivars representing great untapped diversity; which, when bred with modern cultivars can create high yielding and resilient accessions (Ceccarelli et al., 1995; Lakew et al., 1997; Kumar et al., 2020). Several barley collections have been created and introduced previously, but their majority are either global with only a subset of European lines (Liu et al., 2000; Milner et al., 2019), or focus on specific parts of Europe (Igartua et al., 1998; Milotova et al., 2008). Northern European germplasm tends to be under-represented in diversity panels compared to Southern accessions (Pasam et al., 2014; Selçuk et al., 2015). Therefore, there are still aspects of European and in particular Northern European barley diversity that have not been fully explored. The ExHIBiT collection contains many historically important malting varieties from Northern Europe, ranging from landraces to elite cultivars and reflecting the diverse breeding practices used throughout the history of Northern Europe (e.g., Chevalier, RGT Planet, Kenia, Quench, Proctor, Carlsberg (Plarr et al., 1963; Hagenblad & Leino, 2022; Nejat, 2022).

To ensure that the collection’s skew towards Northern European accessions did not introduce a bias and that the genetic diversity of European barley was successfully captured, the ExHIBiT collection was compared to the IPK Barley Core 1000 collection (Milner et al., 2019). IPK Barley Core 1000 is a large collection that aims to capture global barley diversity, including Europe. PCA results of the ExHIBiT collection together with the IPK collection showed that the ExHIBiT accessions fit well with other two-row spring barley accessions present in IPK collection. Specifically, ExHIBiT accessions showed the same diversity to the European two-row spring barley accessions in the IPK collection (**Figure 5**). This result indicates that, the ExHIBiT collection has successfully captured European barley diversity despite its focus on Northern European accessions.

Based on genomic data, population structure of the ExHIBiT collection appears to be mainly defined by the year of release, while the region of origin plays a minimal role. Similar patterns, found in the PCA of the phenotypic data from 2021, further confirms this population structure. This is consistent with results of previous research with non-landrace accessions of European origin (Malysheva-Otto et al., 2006, 2007; Tondelli et al., 2013; Brbaklić et al., 2021). This result contrasts with research focusing on global collections where population structure tends to be defined by country/region of origin (Jones et al., 2011; Muñoz-Amatriaín et al., 2014; Pasam et al., 2014; Russell et al., 2016).

The population structure of the ExHIBiT collection was found to consist of three main groups, which can be broadly described as landraces, pre-1990’s cultivars and post-1990’s released cultivars (**Figure 2**). The ExHIBiT population structure is in good agreement with previous studies focusing on European two-row spring barley. Saade et al. (2020), found that the collection of 377 European two-row spring barley had a population structure made up of three groups, whereas Tondelli et al. (2013), using a collection of 216 European two-row spring barley, found a population structure made up of only two groups. However, the latter collection from Tondelli et al. (2013), does not contain landraces and the two identified groups could be broadly described as pre- and post-1990 released cultivars. The population structure results are consistent with breeding practices in Europe because of the interchange of germplasm between breeding programs, creating a diverse germplasm without major division in the population structure (Rostoks et al., 2006), with most contemporary barley cultivars having four preeminent accessions in their pedigree, these pedigrees being Spratt Archer (from Ireland), Gull (from Sweden), Binder (from Moravia) and Isaria (from Bavaria) (Fischbeck, 1992, 2003, Russell et al., 2000). These results are consistent with the findings of Schreiber et al. (2024), reporting that many European elite barley cultivars are descendant of a small number of “founder” genotypes, namely Kenia, Maja and Gull. The pedigree of the ExHIBiT collection was visualized using the Helium pedigree visualisation platform (Shaw et al., 2014; https://helium.hutton.ac.uk/#/pedigree/exhibit) where along with pedigree definitions, additional characterization data related to the ExHIBiT collection is openly available and can be explored, visualised, and exported should additional downstream analysis be required. These example datasets not only shows the ExHIBiT collection, but also shows where this collection sits in relation to other European barley varieties and how its constituents are related to other varietal material.

Despite the importance of six-row spring barley, the ExHIBiT collection exclusively focuses on two-row spring barley as this is the main type used by the malting industry making it of premium value (Newton et al., 2011; Hertrich, 2013). A small number of the accessions in the collection that were labelled as two-row in their passport data, upon planting were identified as six-row barley. Mislabelling and duplication are the most frequent problems within genebanks, this is due to the large number of accessions maintained (Mascher et al., 2019; Dreiseitl and Nesvadba, 2021). Although the 16 six-row accessions were considered as part of the ExHIBiT collection, they were not considered for inclusion in the core collection. The inclusion of the six-row accessions further supports the idea that the population structure is defined by year of release instead of row-type as breeding history appears to be the most dominant factor. The population structure of the remaining subgroups is not fully explained by row number nor year of release, implying some influence from region of origin or other factors. Malysheva-Otto et al. (2006), found that accessions from Northern Europe and the Soviet Union tend to form subgroups. However, due to the small representation of accessions from some countries and regions, no further assumptions on the reasons behind the structure of the subgroups can be made.

### 4.2 Core collection construction and phenotyping

The core collection with 230 accessions was created to be used in genetic and association mapping studies, effectively reducing the cost and time investment required, while still preserving the diversity of the ExHIBiT collection. One of the main objectives in the establishment of the ExHIBiT core collection was to ensure that the population has sufficient accession numbers with maximum diversity for genetic studies. To create the core collection, a distance-based method called Core Hunter was applied, relying on both genetic and phenotypic data. The choice of appropriate method depended on the purpose of the study (i.e., to capture as much diversity as possible with the smallest number of accessions), in addition to the computational speed of the method, and the information required (i.e., sample size). The distance-based method was ideal as its main purpose was maximising the combination of allelic diversity at genome level, which is a key factor for breeding programs (Leroy et al., 2014). The phenotypic and genotypic diversity in the core and ExHIBiT collection were compared and results showed that the diversity of the whole collection was preserved (**Figures 6** and **7**).

Field data for both ExHIBiT (2020) and core collection (2021) assessment, showed variation in all phenotypic traits, including HEI, FLT, SFM, YLD, TKW and PRO. The checks; RGT Planet, Propino (modern malting barley) and Golden Promise (prominent malting barley in the 1960s). In both years several accessions from the ExHIBiT core collection outperformed the checks in terms of YLD, SFM, HI and PRO. Specifically, Clansman (ID 220), Drost (ID 88), Ladik (ID 253), Primus (ID 260) and Tyne (ID 205) accessions consistently showed superior performance compared to the checks in terms of YLD and HI. Clansman germplasm exhibited exceptional agronomical qualities with consistently higher yields than RGT Planet. Four of these accessions are from Northern Europe (UK, Denmark or Sweden). These results indicate that some of these accessions have a great potential in future breeding programs. However, the ExHIBiT material has not been fully characterised, for biotic and abiotic stresses. The correlation between agronomic performance and year of release is consistent with knowledge of barley breeding priorities, which through time have favoured shorter and early flowering type plants with higher YLD and HI (Monteagudo et al., 2019; Brbaklić et al., 2021).

### 4.3 Association mapping

To confirm the effectiveness of the ExHIBiT collection for future genetic studies, association mapping was performed but not with the aim of identifying new QTLs. Similar validation analysis has been previously carried out by Wang et al. (2021). Due to its high heritability and extensive knowledge, FLT was selected as the validation trait, leading to the identification of significant markers on chromosome 6H and 7H. Previous studies have located several flowering genes on these chromosomes (Alqudah et al., 2014; Bustos-Korts et al., 2019; He et al., 2019). The identified significant SNP marker on the chromosome 6H overlaps with the previously identified flowering gene *HvZTLb* (Bustos-Korts et al., 2019). Furthermore, well-known genes *HvZTLa, Vrn-H3, HvCO8*, and *HvLHY* are in close proximity to the significant SNP markers on chromosome 7H, although they are not situated within the same LD block (Alqudah et al., 2014; Bustos-Korts et al., 2019). The significant marker identified on chromosome 6 (JHI-Hv50k-2016-383902) is also located in close proximity to previously identified QTL in chr6:13,136,770 (Alqudah et al., 2014) that might underlie the earlier described *Eam7* (Stracke and Borrner, 1998). However, some of the most well-described flowering genes including the photoperiod response gene (*Ppd-H1*) and vernalization gene *VRN-H1* (Turner et al., 2005), were not identified in GWAS. This could be explained by previous research suggesting that Northern European barley (which makes up 70% of the ExHIBiT collection) is quite homogenous in terms of flowering genes particularly *Ppd-H1* (Aslan et al., 2015). This observation was confirmed by examining the diversity at SNP markers associated with flowering genes from previous research (Bustos-Korts et al., 2019).

## 5 Conclusion

Barley is an essential crop for food security and has a large genetic diversity, including landraces, old and elite cultivars. In Europe, barley has a high market value with most of its use and growth due to malt processing and breweries. Landraces and old cultivars, which pre-date the Green Revolution represent a great, yet untapped diversity. When bred with elite cultivars, it is possible that high yielding and resilient accessions can be generated. To utilise old accessions in breeding, first their diversity must be explored. The ExHIBiT collection was created to reflect two-row spring European barley diversity, and to be of use in future screening studies and association mapping studies. The ExHIBiT collection provides a better understanding of the genetics of European heritage barley, contributing to improve barley yield, stress resistance, and to promote sustainable barley production in Northern European climates. The public availability of this new, and fully characterised, European heritage collection that contains historically important malting varieties will be useful for breeders, geneticists, physiologists and pathologists around the world, providing a valuable resource for a flourishing malting industry. The 230 ExHIBiT core collection is manageable for field studies and can contribute to the development of barley germplasm as well as to the identification of genomic regions associated with traits of economic importance.

## Data availability statement

The datasets presented in this study can be found in online repositories. The names of the repository/repositories and accession number(s) can be found below: PRJEB67728 (ENA;https://www.ebi.ac.uk/ena/browser/view/PRJEB67728) and Germinate (http://ics.hutton.ac.uk/germinate-exhibit).

## Author contributions

VB: performed field experiments and data collection, analysed and validated genotypic and phenotypic data, performed GWAS and participated in writing of the original draft. NAT: performed field experiments and data collection, analysed and validated genotypic and phenotypic data, performed the power analysis and participated in writing of the original draft. PL: performed field experiments and data collection. GG: performed agronomic design for the field and performed field experiments and data collection. TD: performed field experiments and data collection. JH: performed field experiments and data collection. MH: performed agronomic design for the field. LR: assembled the ExHIBiT collection. KH: assembled the ExHIBiT collection and performed the power analysis. MM: performed genotyping and provided genomic data. PS: assembled pedigree data and created the Germinate databases associated with this work and participated in writing of the original draft. SR: assembled pedigree data and created the Germinate databases associated with this work and participated in writing of the original draft. KM: performed agronomic design for the field. JR assembled the ExHIBiT collection and performed genotyping and provided genomic data. RW: assembled the ExHIBiT collection. MK: analysed and validated genotypic and phenotypic data, performed GWAS, and participated in the writing of the original draft and editing of the manuscript. SN: designed and supervised the project, assembled the ExHIBiT collection, performed field experiments and data collection, analysed and validated genotypic and phenotypic data and reviewed and edited the manuscript. All authors contributed to the review and editing of the manuscript.

## Supplementary material

Supplementary Figure 1 Flow diagram showing genotypic and phenotypic data analysis steps. Single nucleotide polymorphisms (SNP) marker data is available for 363 accessions and 35,968 markers were mapped to ‘Morex’ V3. After quality control (filtering for minor allele frequency of >0.05 and 0.1 individual SNP missing) 9,383 SNP markers were removed. Phenotypic data was lost for 26 genotypes due to unreliable field data and lack of germination, and another 13 genotypes were identified as six-row barley. Genotypic data (26,585 markers) were used for population structure, Principal Component Analysis (PCA) and neighbour joining phylogenetic tree construction. Phenotypic data was used for core selection and genome-wide association study.

Supplementary Figure 2 Phylogenetic tree of the ExHIBiT collection. Neighbour Joining Tree of the 363 accessions in the ExHIBiT collection using 26,585 SNP markers. Bootstrap values are displayed on the branches.

Supplementary Figure 3 Examination of the effect of row type on Principal Component Analysis (PCA) and neighbour joining phylogenetic tree of the ExHIBiT collection **(A)** PCA coloured by row type. **(B)** phylogenetic tree coloured by row type. In purple are shown two-row barley accessions, green represents the six-row barley accessions and pink is used for accessions with unknown row type.

Supplementary Figure 4 Examination of the effect of six subgroups identified in population structure analysis on Principal Component Analysis (PCA) and neighbour joining phylogenetic tree of the ExHIBiT collection **(A)** PCA coloured by six subgroups. **(B)** phylogenetic tree coloured by six subgroups.

Supplementary Figure 5 Diversity panel of ExHIBiT spikes. Selection of spikes from the ExHIBiT spike library to represent the range of phenotypes including the variation in sizes and shapes. Accessions from left to right: Chevalier Tystofte 2, Wisa, Cocktail, Kinnan, Karat, Thuringia, Athos, Alis, Draught, and Beavans 35.

Supplementary Figure 6 Comparison of Principal Component Analysis between the whole ExHIBiT collection and the ExHIBiT core collection. The ExHIBiT core collection is shown in blue and the rest of the ExHIBiT collection in red. The range of values in both data sets have similar values confirming the preservation of genotypic diversity in the selection of the core collection.

Supplementary Figure 7 The Background of the ExHIBiT core collection. **(A)** Breeding history of accessions in the ExHIBiT core collection, divided into landraces, old cultivars (released before 1960) and elite cultivars (released after 1960). **(B)** The decade of release of accessions in the ExHIBiT collection, showing the number of accessions released in each decade in the collection. Landraces are separated as they do not have a specific year of release. **(C)** Country of origin of the accessions in the ExHIBiT core collection. In the case of landraces, this is the country where the accession was found. In the case of the old and elite cultivars, this is the location of the institute or company where breeding took place. The origin of one accession is unknown and four lines originate from outside of Europe.

Supplementary Figure 8 Correlation matrix between phenotypic data for ExHIBiT core collection (from 2021) and ExHIBiT collection (from 2020), both within and between the years. Blue shows positive correlation and red negative correlation. Trait name followed by 20 denotes data from 2020 field trial and trait name followed by 21 denotes data from 2021 field trial.

Supplementary Figure 9 Genome-Wide Association Studies (GWAS) mapping results for Plant Height, Thousand Kernel Weight and Protein content for the ExHIBiT core collection **(A)** Manhattan plot of GWAS analysis using BLINK, with three principal components (PCs) in 2021 Plant Height. In green highlighted significant markers by Bonferroni correction (logarithm of odds (LOD) score of 5.69). In blue is the suggestive line (LOD score of 5) and in red the genome-wide significance line (LOD score of 5.5). **(B)** Boxplot showing phenotypic distribution of plant height of two significant single nucleotide polymorphisms (SNP) identified by GWAS with BLINK model as seen on **(A)** highlighted in green. Red star shows significance according to t-test (p-value< 0.05). **(C)** Manhattan plot of GWAS analysis using BLINK (PC4) in 2021 thousand kernel weight. **(E)** Boxplot showing phenotypic distribution of Thousand Kernel Weight of four significant SNPs identified by GWAS with BLINK model as seen on **(C)** highlighted in green. **(E)** Manhattan plot of GWAS analysis using BLINK (PC4) in 2021 protein content. **(F)** boxplot showing phenotypic distribution of protein content of three significant SNPs identified by GWAS with BLINK model as seen on **(E)** highlighted in green.

## References

[B1] Alqudah A. M. Schnurbusch T. (2017). Heading date is not flowering time in spring barley. Front. Plant Sci. 8. doi: 10.3389/fpls.2017.00896 PMC5447769 28611811

[B2] Alqudah A. M. Sharma R. Pasam R. K. Graner A. Kilian B. Schnurbusch T. (2014). Genetic dissection of photoperiod response based on GWAS of pre-anthesis phase duration in spring barley. PloS One 9, e1131120. doi: 10.1371/journal.pone.0113120 PMC4242610 25420105

[B3] Anscombe F. J. Tukey J. W. (1963). The examination and analysis of residuals. Technometrics 5, 141–160. 10.1080/00401706.1963.10490071

[B4] Araus J. L. Kefauver S. C. Zaman-Allah M. Olsen M. S. Cairns J. E. (2018). Translating high-throughput phenotyping into genetic gain. Trends Plant Sci. 23, 451–466. doi: 10.1016/j.tplants.2018.02.001 29555431 PMC5931794

[B5] Aslan S. Forsberg N. E. G. Hagenblad J. Leino M. W. (2015). Molecular genotyping of historical barley landraces reveals novel candidate regions for local adaption. Crop Sci. 55, 1031–1034. doi: 10.2135/cropsci2015.02.0119

[B6] Badr A. Müller K. Schäfer-Pregl R. el Rabey H. Effgen S. Ibrahim H. H.. (2000). On the origin and domestication history of barley (Hordeum vulgare). Mol. Biol. Evol. 17, 499–510. doi: 10.1093/oxfordjournals.molbev.a026330 10742042

[B7] Bayer M. M. Rapazote-Flores P. Ganal M. W. Hedley P. E. Macaulay M. Plieske J.. (2017). Development and evaluation of a barley 50k iSelect SNP array. Front. Plant Sci. 8. doi: 10.3389/fpls.2017.01792 PMC5651081 29089957

[B8] Berger B. de Regt B. Tester M. (2012). ““High-Throughput Phenotyping of plant shoots,”,” in *High-Throughput Phenotyping in Plants: Methods and Protocols* Methods in Molecular Biology ed. Normanly J. (New York, NY: Humana Press), 9–20. 10.1007/978-1-61779-995-2_2 22893282

[B9] Bradbury P. J. Zhang Z. Kroon D. E. Casstevens T. M. Ramdoss Y. Buckler E. S. (2007). TASSEL: Software for association mapping of complex traits in diverse samples. Bioinformatics 23, 2633–2635. doi: 10.1093/bioinformatics/btm308 17586829

[B10] Brbaklić L. Trkulja D. Mikić S. Mirosavljević M. Momčilović V. Dudić B.. (2021). Genetic diversity and population structure of Serbian barley (Hordeum vulgare l.) collection during a 40-year long breeding period. Agronomy 11, 118. doi: 10.3390/agronomy11010118

[B11] Brien C. (2023) asremlPlus: Augments “ASReml-R” in Fitting Mixed Models and Packages Generally in Exploring Prediction Differences. Available online at: https://CRAN.R-project.org/package=asremlPlus (Accessed March 14, 2023).

[B12] Brown A. H. D. (1989a). Core collections: a practical approach to genetic resources management. Genome 31, 818–824. doi: 10.1139/g89-144

[B13] Brown A. H. D. (1989b). The case for core collections. In Brown A. H. D. (ed.) Use Plant Genet. Resour. Cambridge, England: Cambridge Univ. Press, 136–156.

[B14] Bustos-Korts D. Dawson I. K. Russell J. R. Tondelli A. Guerra D. Ferrandi C.. (2019). Exome sequences and multi-environment field trials elucidate the genetic basis of adaptation in barley. Plant J. 99, 1172–1191. doi: 10.1111/tpj.14414 31108005 PMC6851764

[B15] Butler D. G. Cullis B. R. Gilmour A. R. Gogel B. J. Thompson R. (2017). ASReml-R Reference Manual Version 4. (Hempstead: VSN International Ltd). Available at: http://www.homepages.ed.ac.uk/iwhite/asreml/uop.

[B16] Caldwell K. S. Russell J. R. Langridge P. Powell W. (2006). Extreme population-dependent linkage disequilibrium detected in an inbreeding plant species, Hordeum vulgare. Genetics 172, 557–567. doi: 10.1534/genetics.104.038489 16219791 PMC1456183

[B17] Ceccarelli S. Grando S. Van Leur J. A. G. (1995). Barley landraces of the fertile crescent offer new breeding options for stress environments. Diversity 11, 112–113.

[B18] Champely S. (2020) pwr: Basic Functions for Power Analysis. Available online at: https://CRAN.R-project.org/package=pwr (Accessed March 14, 2023).

[B19] Cullis B. R. Smith A. B. Coombes N. E. (2006). On the design of early generation variety trials with correlated data. J. Agric. Biol. Environ. Stat. 11, 381–393. doi: 10.1198/108571106X154443

[B20] De Beukelaer H. Davenport G. F. Fack V. (2018). Core Hunter 3: Flexible core subset selection. BMC Bioinf. 19, 1–12. doi: 10.1186/s12859-018-2209-z PMC6092719 29855322

[B21] Dreiseitl A. (2021). Genotype heterogeneity in accessions of a winter barley core collection assessed on postulated specific powdery mildew resistance genes. Agronomy 11, 513. doi: 10.3390/agronomy11030513

[B22] Dreiseitl A. Nesvadba Z. (2021). Powdery mildew resistance genes in single-plant progenies derived from accessions of a winter barley core collection. Plants 10, 1–9. doi: 10.3390/plants10101988 PMC8537652 34685797

[B23] Earl D. A. VonHoldt. B. M. (2012). STRUCTURE HARVESTER: a website and program for visualizing STRUCTURE output and implementing the Evanno method. Conserv. Genet. Resour 4, 359–361. doi: 10.1007/s12686-011-9548-7

[B24] Euromalt (2021) Euromalt Statistics. Available online at: https://www.euromalt.be/euromalt-statistics (Accessed June 6, 2023).

[B25] European Commission (2020). A Farm to Fork Strategy for a fair, healthy and environmentally-friendly food system COM/2020/381 final. Communication from the Commission to the European Parliament, the Council, the European Economic and Social Committee and the Committee of the Regions. COM(2020) 381 final. Brussels 20.5.2020. https://eur-lex.europa.eu/legal-content/EN/TXT/?uri=CELEX:52020DC0381. [Accessed 03 March 2024]

[B26] Evanno G. Regnaut S. Goudet J. (2005). Detecting the number of clusters of individuals using the software STRUCTURE: a simulation study. Mol. Ecol. 14, 2611–2620. doi: 10.1111/j.1365-294X.2005.02553.x 15969739

[B27] Faccini N. Delbono S. Oğuz A.Ç. Cattivelli L. Vale G. Tondelli A. (2021). Resistance of european spring 2-row barley cultivars to pyrenophora graminea and detection of associated loci. Agronomy 11, 374. doi: 10.3390/agronomy11020374

[B28] Fernández-Calleja M. Casas A. M. Igartua E. (2021). Major flowering time genes of barley: allelic diversity, effects, and comparison with wheat. Theor. Appl. Genet. 134, 1867–1897. doi: 10.1007/s00122-021-03824-z 33969431 PMC8263424

[B29] Fischbeck G. (1992). Barley Genetics VI: Proceedings of the 6th International Barley Genetics Symposium (Sweden: Helsingborg).

[B30] Fischbeck G. (2003). ““Diversification through breeding,”,” in Diversity in Barley (Hordeum Vulgare). Eds. Bothmer R.v. Hintum T.v. Knuepffer H. Sato K. (Elsevier, Amsterdam), 29–52.

[B31] Fox J. Weisberg S. (2019). An R Companion to Applied Regression Vol. 16 (Vienna: R Foundation for Statistical Computing). Available at: https://socialsciences.mcmaster.ca/jfox/Books/Companion/. Sage.

[B32] Frankel O. H. Brown A. H D. (1984). ““Plant genetic resources today: a critical appraisal,”,” in Crop genetic resources: conservation and evaluation ed. Holden J. H.W. Williams J. T. (London: George Allen and Unwind), 249–257.

[B33] Gilmour A. R. Cullis B. R. Verbyla A. P. (1997). Accounting for natural and extraneous variation in the analysis of field experiments. Source: J. Agricultural Biological Environ. Stat 2, 269–293. doi: 10.2307/1400446

[B34] Guido L. F. (2019). Brewing and craft beer. Beverages 5, 51. doi: 10.3390/beverages5030051

[B35] Hagenblad J. Leino M. W. (2022). Chevalier barley: The influence of a world-leading malting variety. Crop Sci. 62, 235–246. doi: 10.1002/csc2.20668

[B36] Harrell F. E. (2023) Hmisc: Harrell Miscellaneous. Available online at: https://CRAN.R-project.org/package=Hmisc (Accessed March 14, 2023).

[B37] Harwood W. A. (2019). Barley. (Humana New York, NY). doi: 10.1007/978-1-4939-8944-7

[B38] He T. Hill C. B. Angessa T. T. Zhang X. Q. Chen K. Moody D.. (2019). Gene-set association and epistatic analyses reveal complex gene interaction networks affecting flowering time in a worldwide barley collection. J. Exp. Bot. 70, 5603–5616. doi: 10.1093/jxb/erz332 31504706 PMC6812734

[B39] Hertrich (2013). Topics in brewing: malting barley. Tech. Q. 50, 29–41. doi: 10.1094/TQ-50-1-0331-01

[B40] Huang M. Liu X. Zhou Y. Summers R. M. Zhang Z. (2019). BLINK: a package for the next level of genome-wide association studies with both individuals and markers in the millions. Gigascience 8, giy154. doi: 10.1093/gigascience/giy154 30535326 PMC6365300

[B41] Igartua E. Gracia M. P. Lasa J. M. Medina B. Molina-Cano J. L. Montoya J. L.. (1998). The Spanish barley core collection. Genet. Resour Crop Evol. 45, 475–481. doi: 10.1023/A:1008662515059

[B42] Jombart T. Balloux F. Dray S. (2010). adephylo: New tools for investigating the phylogenetic signal in biological traits. Bioinformatics 26, 1907–1909. doi: 10.1093/bioinformatics/btq292 20525823

[B43] Jones H. Civáň P. Cockram J. Leigh F. J. Smith L. M. Jones M. K.. (2011). Evolutionary history of barley cultivation in Europe revealed by genetic analysis of extant landraces. BMC Evolutionary Biol. 11, 1–12. doi: 10.1186/1471-2148-11-320 PMC3248229 22047039

[B44] Khodaeiaminjan M. Knoch D. Ndella T. M. R. Marchetti C. F. Kořínková N. Techer A.. (2023). Genome-wide association study in two-row spring barley landraces identifies QTLs associated with plantlets root system architecture traits in well-watered and osmotic stress conditions. Front. Plant Sci. 14, 1120. doi: 10.3389/fpls.2023.1125672 PMC10106628 37077626

[B45] Korte A. Farlow A. (2013). The advantages and limitations of trait analysis with GWAS: A review. Plant Methods 9, 1–9. doi: 10.1186/1746-4811-9-29 23876160 PMC3750305

[B46] Kristensen P. S. Jahoor A. Andersen J. R. Cericola F. Orabi J. Janss L. L.. (2018). Genome-wide association studies and comparison of models and cross-validation strategies for genomic prediction of quality traits in advanced winter wheat breeding lines. Front. Plant Sci. 9, 69. doi: 10.3389/fpls.2018.00069 29456546 PMC5801407

[B47] Kumar A. Verma R. P. S. Singh A. Kumar Sharma H. Devi G. (2020). Barley landraces: Ecological heritage for edaphic stress adaptations and sustainable production. Environ. Sustainability Indic. 6, 100035. doi: 10.1016/j.indic.2020.100035

[B48] Lakew B. Semeane Y. Alemayehu F. Gebre H. Grando S. Van Leur J. A. G.. (1997). Exploiting the diversity of barley landraces in Ethiopia. Genet. Resour Crop Evol. 44, 109–116. doi: 10.1023/A:1008644901982

[B49] Leroy T. De Bellis F. Legnate H. Musoli P. Kalonji A. Loor Solorzano R. G.. (2014). Developing core collections to optimize the management and the exploitation of diversity of the coffee Coffea canephora. Genetica 142, 185–199. doi: 10.1007/s10709-014-9766-5 24792040

[B50] Liu F. Sun G. L. Salomon B. Von Bothmer R. (2001). Distribution of allozymic alleles and genetic diversity in the American Barley Core Collection. Theor. Appl. Genet. 102, 606–615. doi: 10.1007/s001220051687

[B51] Liu F. Sun G.l. Salomon B. von Bothmer R. (2002). Characterization of genetic diversity in core collection accessions of wild barley, Hordeum vulgare ssp. spontaneum. Hereditas 136, 67–73. doi: 10.1034/j.1601-5223.2002.1360110.x 12184491

[B52] Liu F. von Bothmer R. Salomon B. (1999). Genetic diversity among East Asian accessions of the barley core collection as revealed by six isozyme loci. Theor. Appl. Genet. 98, 1226–1233. doi: 10.1007/s001220051188

[B53] Liu F. Von Bothmer R. Salomon B. (2000). Genetic diversity in European accessions of the Barley Core Collection as detected by isozyme electrophoresis. Genet. Resour Crop Evol. 47, 571–581. doi: 10.1023/A:1026532215990

[B54] Looseley M. E. Ramsay L. Bull H. Swanston J. S. Shaw P. D. Macaulay M.. (2020). Association mapping of malting quality traits in UK spring and winter barley cultivar collections. Theor. Appl. Genet. 133, 2567–2582. doi: 10.1007/s00122-020-03618-9 32506274 PMC7419451

[B55] Malysheva-Otto L. V. Ganal M. W. Law J. R. Reeves J. C. Röder M. S. (2007). Temporal trends of genetic diversity in European barley cultivars (Hordeum vulgare L.). Mol. Breed. 20, 309–322. doi: 10.1007/s11032-007-9093-y

[B56] Malysheva-Otto L. V. Ganal M. W. Röder M. S. (2006). Analysis of molecular diversity, population structure and linkage disequilibrium in a worldwide survey of cultivated barley germplasm (Hordeum vulgare L.). BMC Genet. 7, 1–14. doi: 10.1186/1471-2156-7-6 16433922 PMC1408084

[B57] Marone D. Russo M. A. Mores A. Ficco D. B. M. Laidò G. Mastrangelo A. M.. (2021). Importance of landraces in cereal breeding for stress tolerance. Plants 10, 1267. doi: 10.3390/plants10071267 34206299 PMC8309184

[B58] Mascher M. Schreiber M. Scholz U. Graner A. Reif J. C. Stein N. (2019). Genebank genomics bridges the gap between the conservation of crop diversity and plant breeding. Nat. Genet. 51, 1076–1081. doi: 10.1038/s41588-019-0443-6 31253974

[B59] Mascher M. Wicker T. Jenkins J. Plott C. Lux T. Koh C. S.. (2021). Long-read sequence assembly: a technical evaluation in barley. Plant Cell 33, 1888–1906. doi: 10.1093/plcell/koab077 33710295 PMC8290290

[B60] Masson-Delmotte V. Zhai P. Pirani A. Connors S. L. Péan C. Berger S.. (2021). IPCC 2021: Climate Change 2021: The Physical Science Basis (New York, NY, USA: Cambridge University Press, Cambridge, United Kingdom).

[B61] Maxted N. Scholten M. Ford-Lloyd B. Allender C. Astley D. Vincent H.. (2014). Landrace conservation strategy for the United Kingdom (Birmingham, UK: The University of Birmingham).

[B62] Milner S. G. Jost M. Taketa S. Mazón E. R. Himmelbach A. Oppermann M.. (2019). Genebank genomics highlights the diversity of a global barley collection. Nat. Genet. 51, 319–326. doi: 10.1038/s41588-018-0266-x 30420647

[B63] Milotova J. Martynov S. P. Dobrotvorskaya T. V. Vaculova K. (2008). Genealogical analysis of the diversity of spring barley cultivars released in former CzechoSlovakia and modern Czech Republic. Russ J. Genet. 44, 51–59. doi: 10.1134/S1022795408010079 18409388

[B64] Monteagudo A. Casas A. M. Cantalapiedra C. P. Contreras-Moreira B. Gracia M. P. Igartua E. (2019). Harnessing novel diversity from landraces to improve an elite barley variety. Front. Plant Sci. 10. doi: 10.3389/fpls.2019.00434 PMC6470277 31031782

[B65] Muñoz-Amatriaín M. Cuesta-Marcos A. Endelman J. B. Comadran J. Bonman J. M. Bockelman H. E.. (2014). The USDA barley core collection: Genetic diversity, population structure, and potential for genome-wide association studies. PloS One 9, 1–13. doi: 10.1371/journal.pone.0094688 PMC3986206 24732668

[B66] Nejat N. (2022). Gene editing of the representative WRKY family members in an elite malting barley cultivar RGT Planet by CRISPR/Cas9. Diss. Murdoch University. doi: 10.13140/RG.2.2.20399.10408

[B67] Newton A. C. Flavell A. J. George T. S. Leat P. Mullholland B. Ramsay L.. (2011). Crops that feed the world 4. Barley: a resilient crop? Strengths and weaknesses in the context of food security. Food Secur 3, 141–178. doi: 10.1007/s12571-011-0126-3

[B68] Newton A. C. Gravouil C. Fountaine J. M. (2010). Managing the ecology of foliar pathogens: Ecological tolerance in crops. Ann. Appl. Biol 157, 343–359. doi: 10.1111/j.1744-7348.2010.00437.x

[B69] Nolan P. Flanagan J. (2022). High-resolution climate projections for Ireland - A multi-model ensemble approach. Available from: https://www.epa.ie/publications/research/climate-change/research-339-high-resolution-climate-projections-for-ireland-.php. [Accessed 3rd March 2024]

[B70] Paradis E. Claude J. Strimmer K. (2004). APE: Analyses of phylogenetics and evolution in R language. Bioinformatics 20, 289–290. doi: 10.1093/bioinformatics/btg412 14734327

[B71] Pasam R. K. Sharma R. Walther A. Özkan H. Graner A. Kilian B. (2014). Genetic diversity and population structure in a legacy collection of spring barley landraces adapted to a wide range of climates. PloS One 9, e116164. doi: 10.1371/journal.pone.0116164 25541702 PMC4277474

[B72] Plarr W. Hoffmann W. Göpp K. Broekhuizen S. (1963). “Barley growing and breeding in Europe,” in Broekhuizen S. (Ed.), International Barley Genetics Symposium, Wageningen.

[B73] Pritchard J. K. Stephens M. Donnelly P. (2000). Inference of population structure using multilocus genotype data. Genetics 155, 945–959. doi: 10.1093/genetics/155.2.945 10835412 PMC1461096

[B74] Prosekov A. Y. Ivanova S. A. (2018). Food security: The challenge of the present. Geoforum 91, 73–77. doi: 10.1016/j.geoforum.2018.02.030

[B75] Raubach S. Kilian B. Dreher K. Amri A. Bassi F. M. Boukar O.. (2021). From bits to bites: Advancement of the Germinate platform to support prebreeding informatics for crop wild relatives. Crop Sci. 61, 1538–1566. doi: 10.1002/csc2.20248

[B76] R Core Team (2022) R: A Language and Environment for Statistical Computing. Available online at: https://www.R-project.org/ (Accessed November 22, 2022).

[B77] Revell L. J. (2012). phytools: An R package for phylogenetic comparative biology (and other things). Methods Ecol. Evol. 3, 217–223. doi: 10.1111/j.2041-210X.2011.00169.x

[B78] Riedelsheimer C. Lisec J. Czedik-Eysenberg A. Sulpice R. Flis A. Grieder C.. (2012). Genome-wide association mapping of leaf metabolic profiles for dissecting complex traits in maize. Proc. Natl. Acad. Sci. 109, 8872–8877. doi: 10.1073/pnas.1120813109 22615396 PMC3384205

[B79] Rostoks N. Ramsay L. MacKenzie K. Cardle L. Bhat P. R. Roose M. L.. (2006). Recent history of artificial outcrossing facilitates whole-genome association mapping in elite inbred crop varieties. Proc. Natl. Acad. Sci. 103, 18656–18661. doi: 10.1073/pnas.0606133103 17085595 PMC1693718

[B80] Russell J. R. Ellis R. P. Thomas W. T. Waugh R. Provan J. Booth A.. (2000). A retrospective analysis of spring barley germplasm development from foundation genotypes’ to currently successful cultivars. Mol. Breed. 6, 553–568. doi: 10.1023/A:1011372312962

[B81] Russell J. Mascher M. Dawson I. K. Kyriakidis S. Calixto C. Freund F.. (2016). Exome sequencing of geographically diverse barley landraces and wild relatives gives insights into environmental adaptation. Nat. Genet. 48, 1024–1030. doi: 10.1038/ng.3612 27428750

[B82] Saade S. Brien C. Pailles Y. Berger B. Shahid M. Russell J.. (2020). Dissecting new genetic components of salinity tolerance in two-row spring barley at the vegetative and reproductive stages. PloS One 15, 1–19. doi: 10.1371/journal.pone.0236037 PMC7377408 32701981

[B83] Saade S. Maurer A. Shahid M. Oakey H. Schmöckel S. M. Negrão S.. (2016). Yield-related salinity tolerance traits identified in a nested association mapping (NAM) population of wild barley. Sci. Rep. 6, 1–9. doi: 10.1038/srep32586 27585856 PMC5009332

[B84] Saitou N. Nei M. (1987). The neighbor-joining method: a new method for reconstructing phylogenetic trees. Mol. Biol. Evol. 4, 406–425. 3447015 10.1093/oxfordjournals.molbev.a040454

[B85] Sasidharan R. Voesenek L. A. C. J. Perata P. (2021). Plant performance and food security in a wetter world. New Phytol. 229, 5–7. doi: 10.1111/nph.17067 33285019

[B86] Schmidt S. B. Brown L. K. Booth A. Wishart J. Hedley P. E. Martin P.. (2023). Heritage genetics for adaptation to marginal soils in barley. Trends Plant Sci. 28, 544–551. doi: 10.1016/j.tplants.2023.01.008 36858842

[B87] Schmidt S. B. George T. S. Brown L. K. Booth A. Wishart J. Hedley P. E.. (2019). Ancient barley landraces adapted to marginal soils demonstrate exceptional tolerance to manganese limitation. Ann. Bot. 123, 831–843. doi: 10.1093/aob/mcy215 30561497 PMC6526322

[B88] Schreiber M. Wonneberger R. Haaning A. M. Coulter M. Russell J. Himmelbach A.. (2024). Genomic resources for a historical collection of cultivated two-row European spring barley genotypes. Sci Data 11, 66. doi: 10.1038/s41597-023-02850-4 38216606 PMC10786862

[B89] Selçuk A. Forsberg N. Hagenblad J. Leino M. W. (2015). Molecular genotyping of historical barley landraces reveals novel candidate regions for local adaption. Crop Sci. 55, 2766–2776. doi: 10.2135/cropsci2015.02.0119

[B90] Shaw P. D. Graham M. Kennedy J. Milne I. Marshall D. F. (2014). Helium: visualization of large scale plant pedigrees. BMC Bioinf. 15, 1–15. doi: 10.1186/1471-2105-15-259 PMC4133633 25085009

[B91] Shin J.-H. Blay S. McNeney B. Graham J. (2006). LDheatmap: an R function for graphical display of pairwise linkage disequilibria between single nucleotide polymorphisms. J. Stat. Soft 16, 1–9. doi: 10.18637/jss.v016.c03

[B92] Slama A. Mallek-Maalej E. Mohamed H. Rhim T. Radhouane L. (2018). A return to the genetic heritage of durum wheat to cope with drought heightened by climate change. PloS One 13, e0196873. doi: 10.1371/journal.pone.0196873 29795584 PMC5967785

[B93] Sleight J. (2022)Cutting emissions in malting barley by 50% in five years. In: The Scottish Farmer. Available online at: https://www.thescottishfarmer.co.uk/news/23165268.cutting-emissions-malting-barley-50-five-years/ (Accessed June 6, 2023).

[B94] Stacklies W. Redestig H. Scholz M. Walther D. Selbig J. (2007). pcaMethods - A bioconductor package providing PCA methods for incomplete data. Bioinformatics 23, 1164–1167. doi: 10.1093/bioinformatics/btm069 17344241

[B95] Storey J. D. Bass A. J. Dabney A. Robinson D. (2022) qvalue: Q-value estimation for false discovery rate control. Available online at: http://github.com/jdstorey/qvalue.

[B96] Storey J. D. Tibshirani R. (2003). Statistical significance for genomewide studies. Proc. Natl. Acad. Sci. 100, 9440–9445. doi: 10.1073/pnas.1530509100 12883005 PMC170937

[B97] Stracke S. Borrner A. (1998). Molecular mapping of the photoperiod response gene ea7 in barley. Theor. Appl. Genet. 97, 797–800. doi: 10.1007/s001220050958

[B98] Tanksley S. D. Mccouch S. R. (1997). Seed banks and molecular maps: unlocking genetic potential from the wild the narrow genetic base of crop plants. Sci. (1979) 277, 1063–1066. doi: 10.1126/science.277.5329.1063 9262467

[B99] Tondelli A. Xu X. Moragues M. Sharma R. Schnaithmann F. Ingvardsen C.. (2013). Structural and temporal variation in genetic diversity of European spring two-row barley cultivars and association mapping of quantitative traits. Plant Genome 6, 1–14. doi: 10.3835/plantgenome2013.03.0007

[B100] Turner A. Beales J. Faure S. Dunford R. P. Laurie D. A. (2005). The pseudo-response regulator Ppd-H1 provides adaption to photoperiod in barley. Sci. (1979) 11, 1031–1034. doi: 10.1126/science.1117682 16284181

[B101] Uleberg E. Hanssen-Bauer I. van Oort B. Dalmannsdottir S. (2014). Impact of climate change on agriculture in Northern Norway and potential strategies for adaptation. Clim Change 122, 27–39. doi: 10.1007/s10584-013-0983-1

[B102] Umego E. C. Barry-Ryan C. (2022). Overview of the Irish brewing and distilling sector: Processing inputs supply and quality requirements. BrewingScience 75, 9–16. doi: 10.23763/BrSc21-19umego

[B103] van Hintum T. J.L. (1994). Comparison of marker systems and construction of a core collection in a pedigree of European spring barley. Theor. Appl. Genet. 89, 991–997. doi: 10.1007/BF00224529 24178115

[B104] van Treuren R. Tchoudinova I. van Soest L. J. M. van Hintum T. J. L. (2006). Marker-assisted acquisition and core collection formation: A case study in barley using AFLPs and pedigree data. Genet. Resour Crop Evol. 53, 43–52. doi: 10.1007/s10722-004-0585-x

[B105] Wang X. Ando K. Wu S. Reddy U. K. Tamang P. Bao K.. (2021). Genetic characterization of melon accessions in the U.S. National Plant Germplasm System and construction of a melon core collection. Mol. Horticulture 1, 1–13. doi: 10.1186/s43897-021-00014-9 PMC10515074 37789496

[B106] Wang J. Zhang Z. (2021). GAPIT version 3: boosting power and accuracy for genomic association and prediction. Genomics Proteomics Bioinf. 19, 629–640. doi: 10.1016/j.gpb.2021.08.005 PMC9121400 34492338

[B107] Wei T. Simko V. (2021) Package “corrplot”: Visualization of a Correlation Matrix. Available online at: https://github.com/taiyun/corrplot (Accessed March 14, 2023).

[B108] Weise S. Oppermann M. Maggioni L. Van Hintum T. Knupffer H. (2017). EURISCO: The European search catalogue for plant genetic resources. Nucleic Acids Res. 45, D1003–D1008. doi: 10.1093/nar/gkw755 27580718 PMC5210606

[B109] Wickham H. (2016) ggplot2: Elegant Graphics for Data Analysis (New York: Springer-Verlag). Available online at: https://ggplot2.tidyverse.org (Accessed March 14, 2023).

[B110] Xie W. Xiong W. Pan J. Ali T. Cui Q. Guan D.. (2018). Decreases in global beer supply due to extreme drought and heat. Nat. Plants 4, 964–973. doi: 10.1038/s41477-018-0263-1 30323183

[B111] Zadoks J. C. Chang T. T. Konzak C. F. (1974). A decimal code for the growth stages of cereals. Weed Res. 14, 415–421. doi: 10.1111/j.1365-3180.1974.tb01084.x

