# Supplementary figures and images for: Unlocking the genetic diversity and population structure of the newly introduced two-row spring European HerItage Barley collecTion (ExHIBiT)
Source: Front Plant Sci. 2024 Mar 20;15:1268847. doi: 10.3389/fpls.2024.1268847 (PMC10987740; doi:10.3389/fpls.2024.1268847)

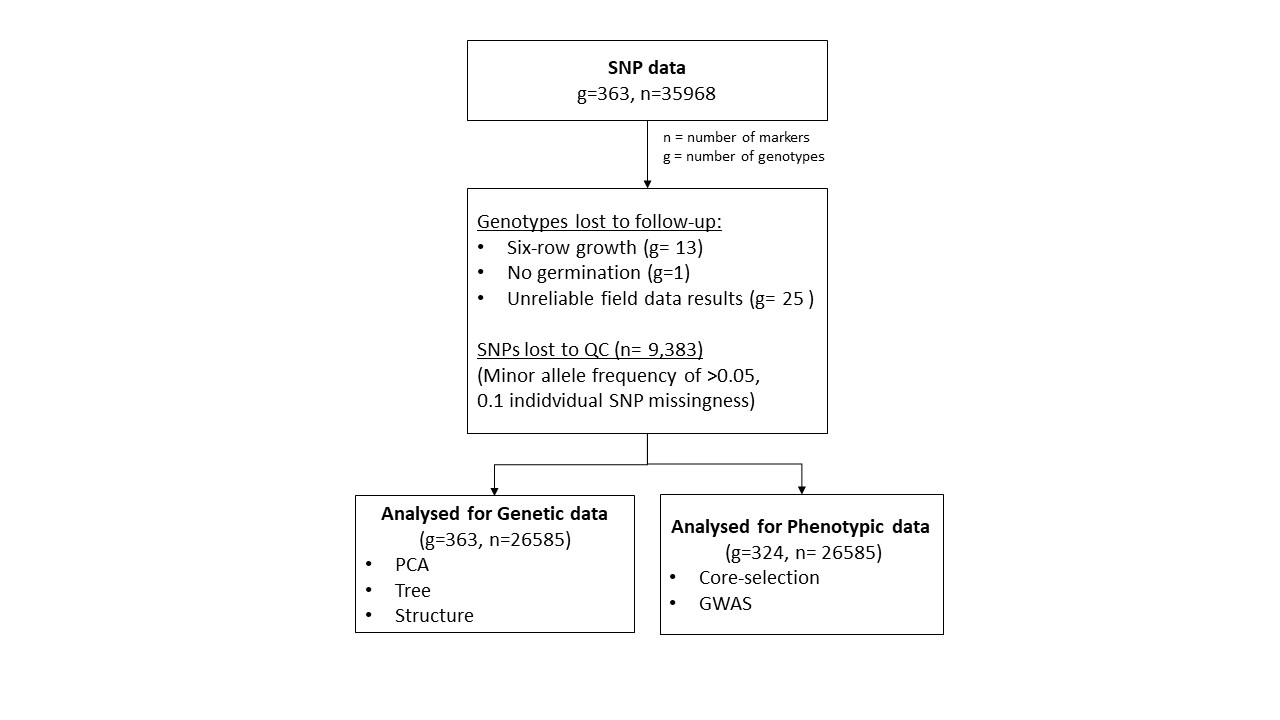

Supplement: Supplementary Figure 1 — Flow diagram showing genotypic and phenotypic data analysis steps. Single nucleotide polymorphisms (SNP) marker data is available for 363 accessions and 35,968 markers were mapped to ‘Morex’ V3. After quality control (filtering for minor allele frequency of >0.05 and 0.1 individual SNP missing) 9,383 SNP markers were removed. Phenotypic data was lost for 26 genotypes due to unreliable field data and lack of germination, and another 13 genotypes were identified as six-row barley. Genotypic data (26,585 markers) were used for population structure, Principal Component Analysis (PCA) and neighbour joining phylogenetic tree construction. Phenotypic data was used for core selection and genome-wide association study. [file Image_1.jpeg]

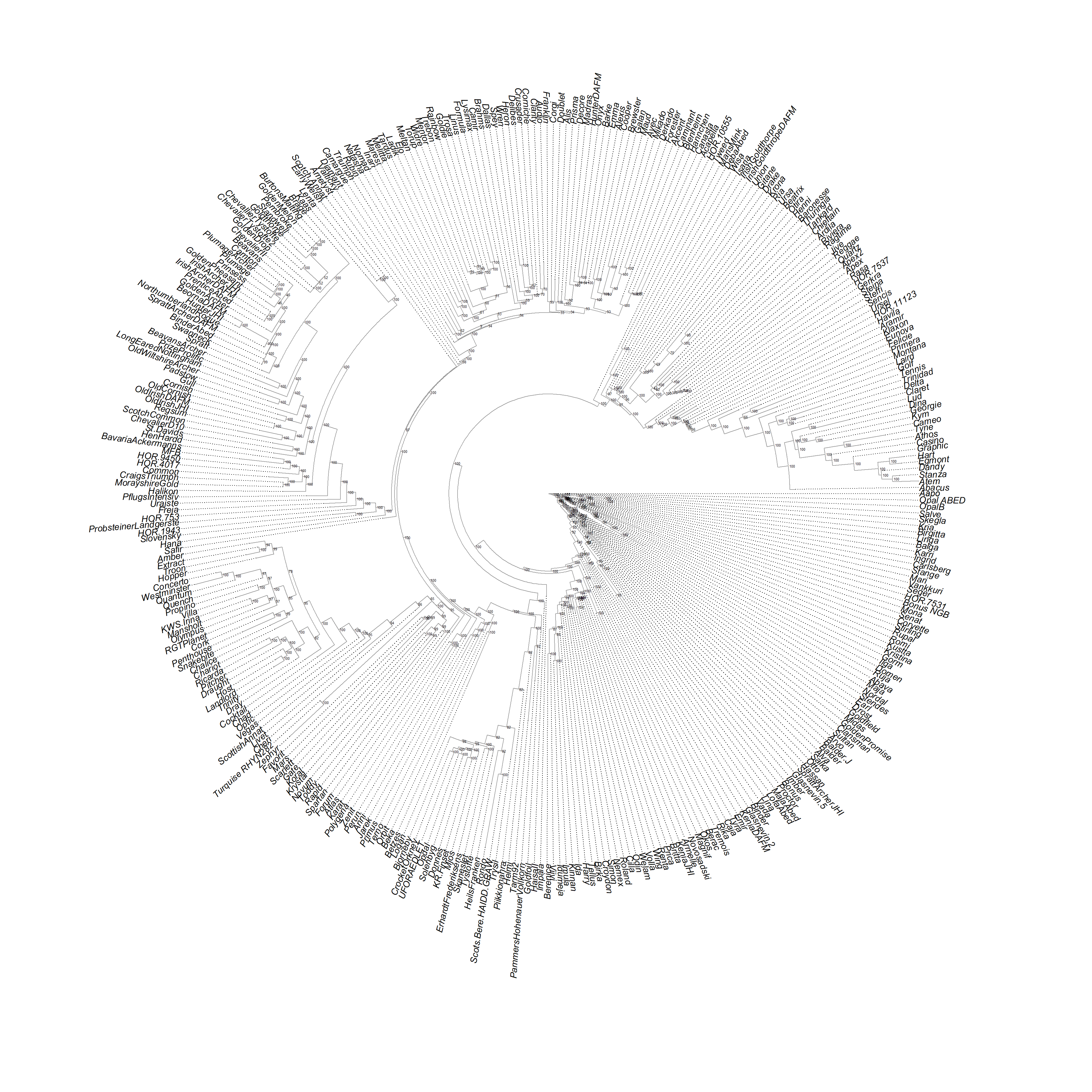

Supplement: Supplementary Figure 2 — Phylogenetic tree of the ExHIBiT collection. Neighbour Joining Tree of the 363 accessions in the ExHIBiT collection using 26,585 SNP markers. Bootstrap values are displayed on the branches. [file Image_2.png]

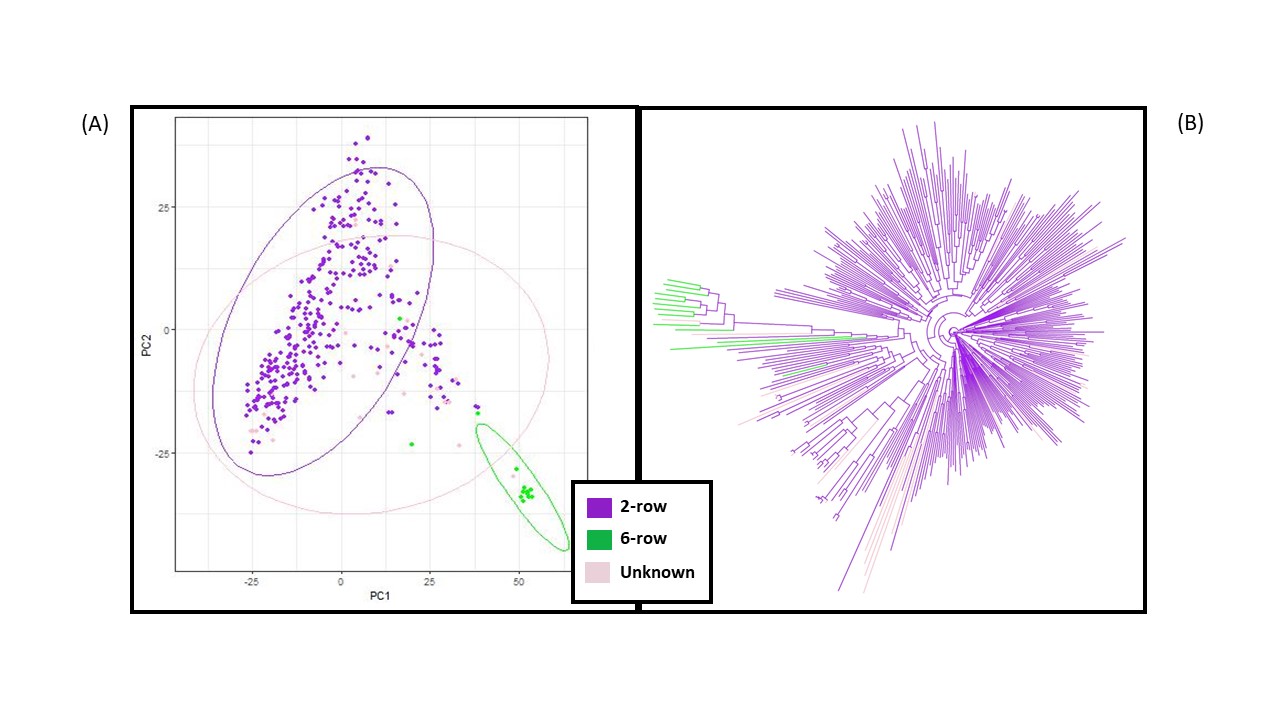

Supplement: Supplementary Figure 3 — Examination of the effect of row type on Principal Component Analysis (PCA) and neighbour joining phylogenetic tree of the ExHIBiT collection (A) PCA coloured by row type. (B) phylogenetic tree coloured by row type. In purple are shown two-row barley accessions, green represents the six-row barley accessions and pink is used for accessions with unknown row type. [file Image_3.jpeg]

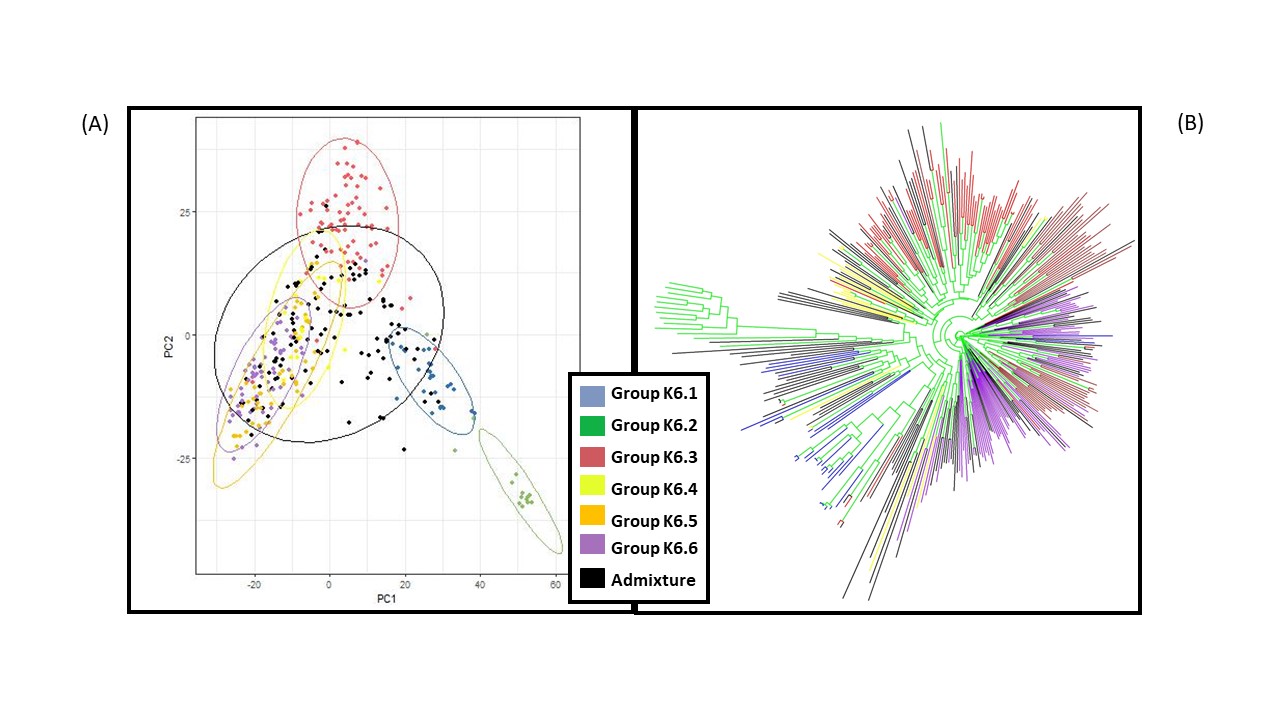

Supplement: Supplementary Figure 4 — Examination of the effect of six subgroups identified in population structure analysis on Principal Component Analysis (PCA) and neighbour joining phylogenetic tree of the ExHIBiT collection (A) PCA coloured by six subgroups. (B) phylogenetic tree coloured by six subgroups. [file Image_4.jpeg]

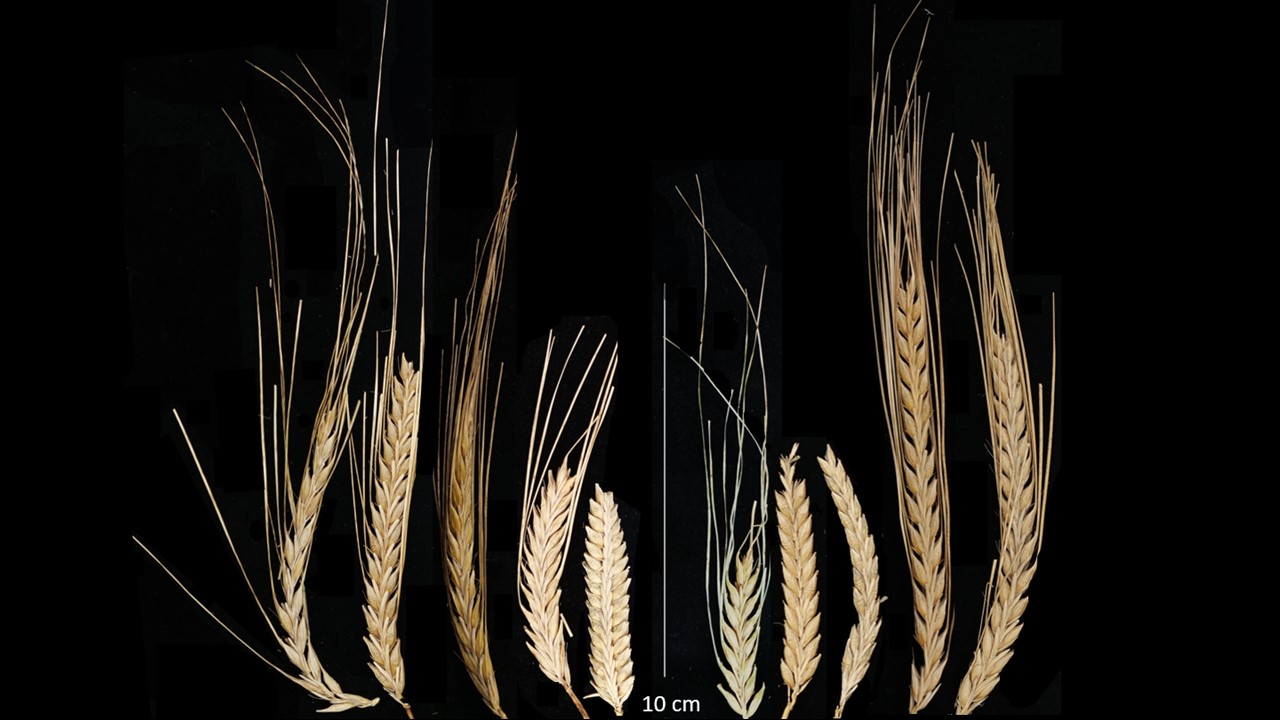

Supplement: Supplementary Figure 5 — Diversity panel of ExHIBiT spikes. Selection of spikes from the ExHIBiT spike library to represent the range of phenotypes including the variation in sizes and shapes. Accessions from left to right: Chevalier Tystofte 2, Wisa, Cocktail, Kinnan, Karat, Thuringia, Athos, Alis, Draught, and Beavans 35. [file Image_5.jpeg]

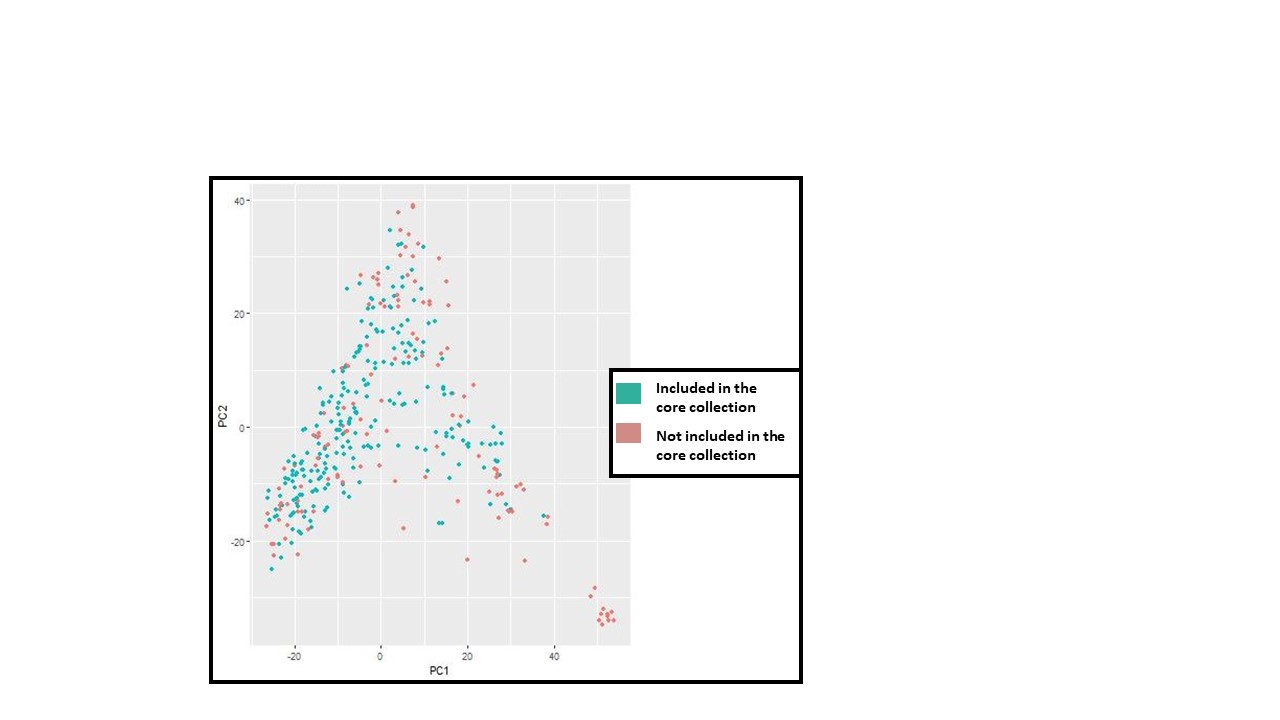

Supplement: Supplementary Figure 6 — Comparison of Principal Component Analysis between the whole ExHIBiT collection and the ExHIBiT core collection. The ExHIBiT core collection is shown in blue and the rest of the ExHIBiT collection in red. The range of values in both data sets have similar values confirming the preservation of genotypic diversity in the selection of the core collection. [file Image_6.jpeg]

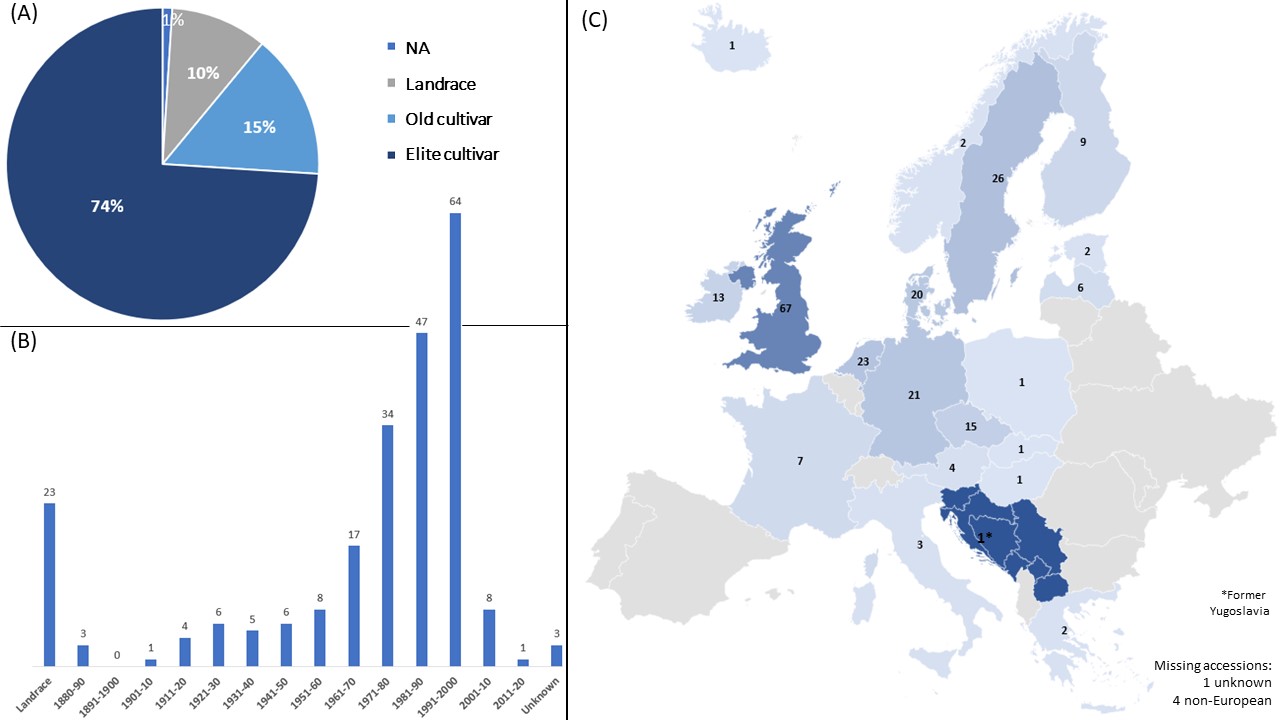

Supplement: Supplementary Figure 7 — The Background of the ExHIBiT core collection. (A) Breeding history of accessions in the ExHIBiT core collection, divided into landraces, old cultivars (released before 1960) and elite cultivars (released after 1960). (B) The decade of release of accessions in the ExHIBiT collection, showing the number of accessions released in each decade in the collection. Landraces are separated as they do not have a specific year of release. (C) Country of origin of the accessions in the ExHIBiT core collection. In the case of landraces, this is the country where the accession was found. In the case of the old and elite cultivars, this is the location of the institute or company where breeding took place. The origin of one accession is unknown and four lines originate from outside of Europe. [file Image_7.jpeg]

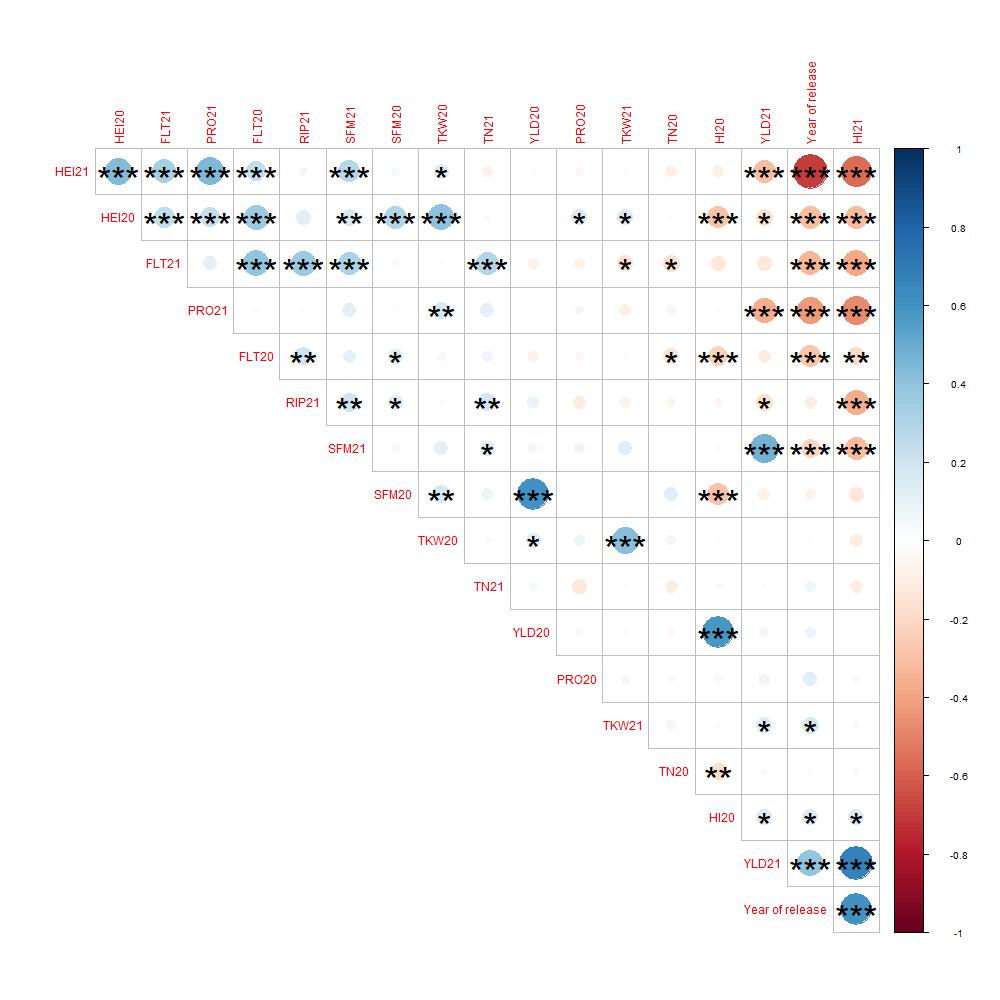

Supplement: Supplementary Figure 8 — Correlation matrix between phenotypic data for ExHIBiT core collection (from 2021) and ExHIBiT collection (from 2020), both within and between the years. Blue shows positive correlation and red negative correlation. Trait name followed by 20 denotes data from 2020 field trial and trait name followed by 21 denotes data from 2021 field trial. [file Image_8.jpeg]

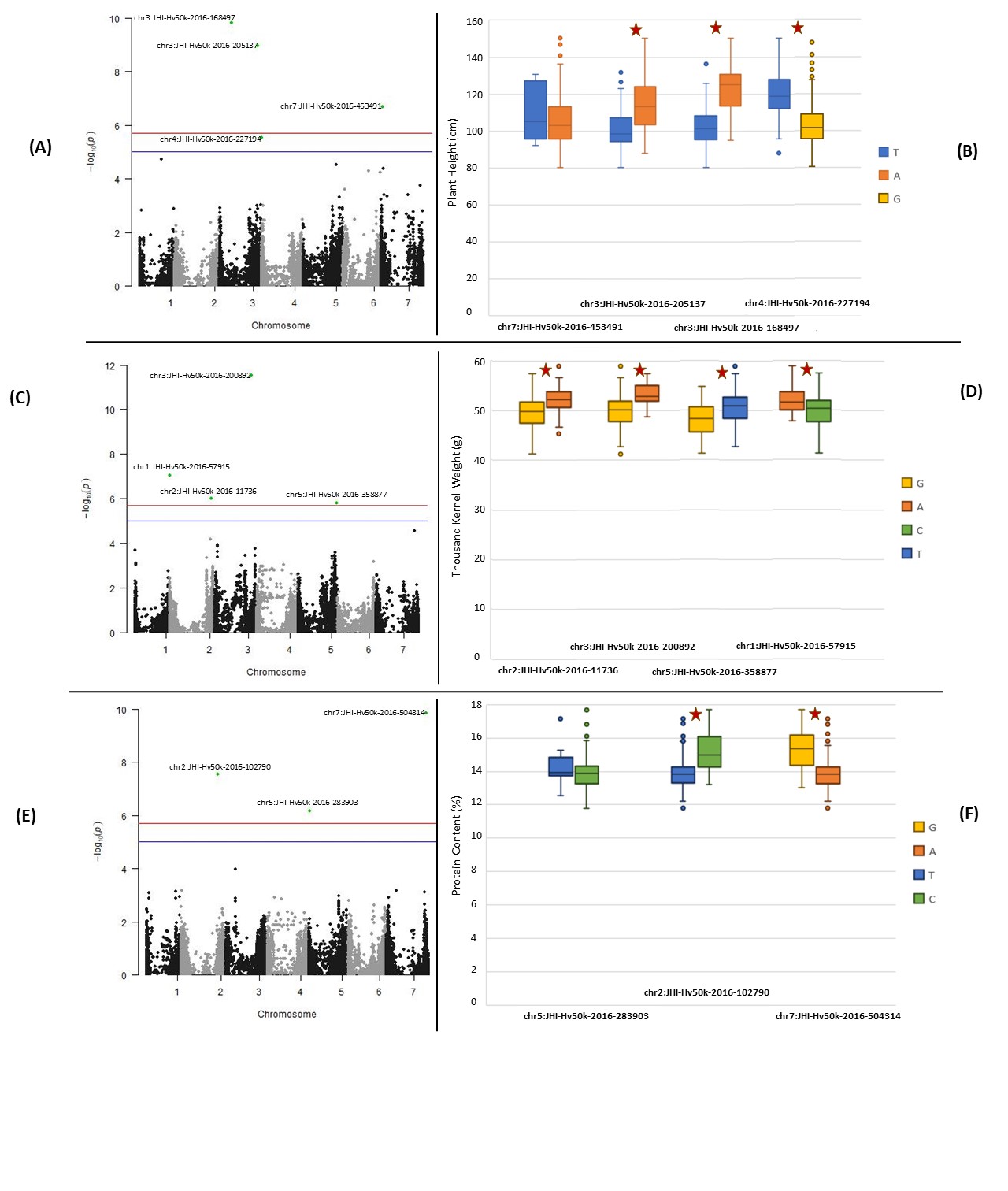

Supplement: Supplementary Figure 9 — Genome-Wide Association Studies (GWAS) mapping results for Plant Height, Thousand Kernel Weight and Protein content for the ExHIBiT core collection (A) Manhattan plot of GWAS analysis using BLINK, with three principal components (PCs) in 2021 Plant Height. In green highlighted significant markers by Bonferroni correction (logarithm of odds (LOD) score of 5.69). In blue is the suggestive line (LOD score of 5) and in red the genome-wide significance line (LOD score of 5.5). (B) Boxplot showing phenotypic distribution of plant height of two significant single nucleotide polymorphisms (SNP) identified by GWAS with BLINK model as seen on (A) highlighted in green. Red star shows significance according to t-test (p-value< 0.05). (C) Manhattan plot of GWAS analysis using BLINK (PC4) in 2021 thousand kernel weight. (E) Boxplot showing phenotypic distribution of Thousand Kernel Weight of four significant SNPs identified by GWAS with BLINK model as seen on (C) highlighted in green. (E) Manhattan plot of GWAS analysis using BLINK (PC4) in 2021 protein content. (F) boxplot showing phenotypic distribution of protein content of three significant SNPs identified by GWAS with BLINK model as seen on (E) highlighted in green. [file Image_9.jpeg]
